# Construction of highly-stable covalent organic framework with combined enol-imine and keto-enamine linkages†

**DOI:** 10.1039/d3ra02251j

**Published:** 2023-05-15

**Authors:** Jian Jiang, Kaiyu He, Xue Cai, Hong Yu, Minghui Zuo, Guan Yun, Tao Yun, Yue Ma, Zitong Zhang, Yunling Liu, Zhenlu Wang

**Affiliations:** a Heilongjiang Key Laboratory of Photoelectric Functional Materials, College of Chemistry and Chemical Engineering, Mudanjiang Normal University Mudanjiang 157011 P. R. China jianjiang@mdjnu.edu.cn; b State Key Laboratory of Photocatalysis on Energy and Environment, College of Chemistry, Fuzhou University Fuzhou 350116 P. R. China; c College of Chemistry, Jilin University 2519 Jiefang Road Changchun 130021 P. R. China

## Abstract

A novel covalent organic framework (COF) (Tp-BI-COF) with combined ketimine-type enol-imine and keto-enamine linkages was prepared through a cascade of ketimine condensation followed by aldimine condensation and characterized by XRD, solid state ^13^C NMR, IR, TGA and BET. Tp-BI-COF showed high stability toward acid, organic solvent, and boiling water. The 2D COF exhibited photochromic properties after being irradiated with a xenon lamp. The stable COF, with aligned one-dimensional nanochannels, provided nitrogen sites on pore walls, which confine and stabilize the H_3_PO_4_ in the channel *via* hydrogen-bonding interactions. After loading with H_3_PO_4_, the material showed excellent anhydrous proton conductivity.

## Abstract 2

A COF with both keto-enamine and ketimine linkages is reported for the first time, which shows high chemical and thermal stability.

## Graphical abstract

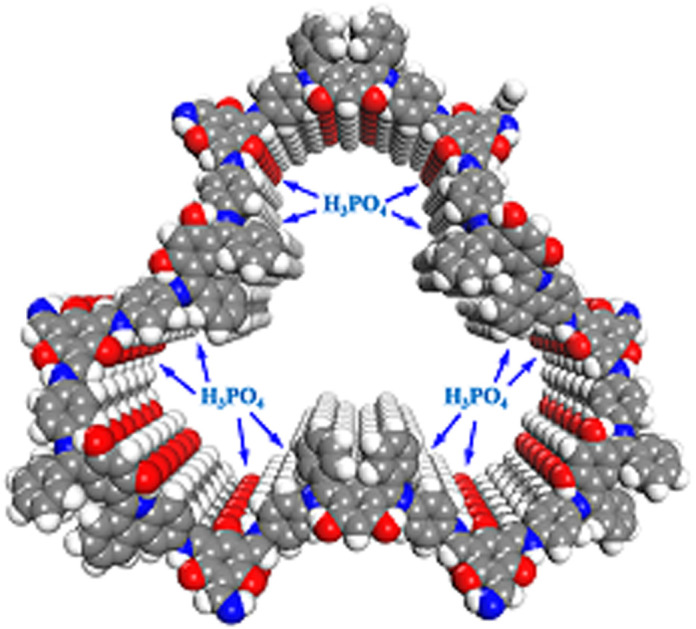

## Introduction

Covalent organic frameworks (COFs) are porous crystalline polymers with well-defined frameworks and ordered pores,^1^ which can be constructed through covalent bonds among elements such as C, N, O, B and Si.^2^ The unique crystallinity of COFs is thought to be provided by the reversibility of covalent bonds formed during synthesis,^3^ for example, reversible formation of borate, borazine, imine, triazine, and hydrazone bonds resulting in a highly crystalline framework.^4^ The most attractive feature of COFs is that their structure, including the size and shape of the frameworks and pores, can be precisely controlled by changing their building blocks and linkages.^5^ In particular, 2D COFs integrate organic building blocks into covalent 2D sheets and frameworks to form periodic arrays and directional open nanochannels. To date, 2D COFs have emerged as powerful platforms for designing functional materials with catalytic,^6^ photoconductive,^7^ gas storage,^8^ charge transport^9^ and photovoltaic properties.^10^

Due to the reversible reactions that occur after COFs are synthesized, in the presence of ambient humidity, COFs usually decompose.^11^ Therefore, the instability of covalent organic frameworks remains a difficult problem, preventing COFs from being widely used in various practical applications. Because of irreversible nature of keto-enamine linkage, it has been used to construct COFs with exceptional stability.^4*a*^ As one of the most common products of Schiff-base condensation, ketimine has been proved to be much more kinetically inert than aldimine,^12^ which make it a good candidate to compose stable COFs. However, as far as we know, few COFs were constructed with ketimine as linkage in literature.

**Fig. 1:**
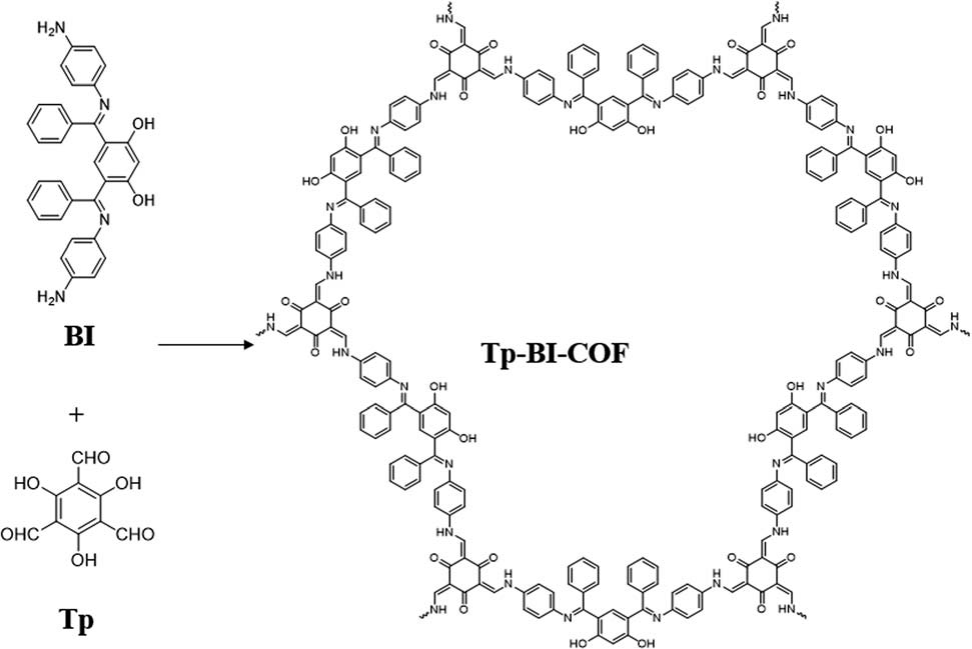
Schematic representation of the synthesis of Tp-BI-COF.

Here, we report the synthesis of a novel 2D COF with cloverleaf-shaped pore, Tp-BI-COF, through a cascade of ketimine condensation followed by aldimine condensation. The aldimine formed suffered irreversible enol-to-keto tautomerization to obtain keto-enamine linkage. Tp-BI-COF contains both keto-enamine and ketimine linkage, which give it excellent chemical and thermal stability (Fig. 1).

## Experimental section

### General

Unless stated otherwise all reagents were purchased from commercial source and used without purification. 1,3,5-Triformylphloroglucinol^13^ and 1,3-dihydroxy-4,6-dibenzoylbenzen^14^ (Tp) was synthesized according to literature methods. P-phenylenediamine was purified by sublimation before use.

### Measurements

X-ray powder diffraction (PXRD) patterns were recorded on a Rigaku Ultima IV-185 diffractometer using Cu Kα radiation (*λ* = 1.54056 Å) over the 2*θ* range of 1.8°–30° and on a SAXSess mc2 diffractometer using Cu Kα radiation (*λ* = 1.54056 Å) over the 2*θ* range of 0.5°–5°. The Shimadzu UV-2600 UV-Vis spectrophotometer, equipped with integration sphere ISR-2200, was used to measure the UV-Vis diffuse reflectance spectrum of solid powders at room temperature. FT-IR spectra were measured on a PerkinElmer Frontier infrared spectrometer FTIR-650 ranging from 4000 to 400 cm^−1^. The thermogravimetric analysis was performed on a Netzsch TG 209 F1 Libra thermogravimetric analyzer with a ramp rate of 10 K min^−1^ and a temperature of 35 to 800 °C under the atmosphere of nitrogen. Elemental analysis (C, H, O and N) was performed on a PerkinElmer 2400chn elemental analyzer. SEM images were obtained using a JSM-6480LV at 5.0 kV. FEI (Jeol FEG 2100F) high resolution transmission electron microscope (HRTEM) equipped with field emission source operating at 300 kV was used to record TEM images. The nitrogen adsorption and desorption isotherms were measured at 77 K using an Autosorb-iQ (Quantachrome) surface area size analyzer. Before measurement, the samples were degassed in vacuum at 100 °C for 10 h. The Brunauer–Emmett–Teller (BET) method was utilized to calculate the specific surface area. ^1^H and ^13^CNMR spectrum were measured on a Bruker Fourier 400 MHz spectrometer. Solid state cross polarization magic angle spinning (CP/MAS) NMR was taken on an AVIII 500 MHz solid-state NMR spectrometer. Mass spectra were obtained on the SolariX XR-15T FTMS Mass Spectrometry Facility. X-ray photoelectron spectroscopy (XPS) was measured on the Thermo ESCALAB 250XI instrument and the C 1s line at 284.8 eV was used as the binding energy reference.

### Preparation of triformylphloroglucinol (Tp)

Prepared as previously reported.^13^ To hexamethylenetetraamine (15.098 g, 108 mmol) and dried phloroglucinol (6.014 g, 49 mmol) was added 90 ml trifluoroacetic acid under N_2_. The solution was heated at 100 °C for *ca.* 2.5 h. Approximately 150 ml of 3 M HCl was added and the solution was heated at 100 °C for 1 h. After cooling to room temperature, the solution was filtered through Celite, extracted with *ca.* 600 ml dichloromethane, dried over magnesium sulfate, and filtered. Rotary evaporation of the solution afforded 1.25 g (11.82%) of an off-white powder. ^1^H NMR indicated near 99% purity; a pure sample was obtained by sublimation. ^1^H NMR (400 MHz, CDCl_3_): *δ* 14.11 (s, 3H), 10.15 (s, 3H) ppm. MP = 198–200 °C. *ρ* = 1.755 g cm^−3^. Solubility: CH_2_Cl_2_ (Slightly, Heated), DMSO (slightly, heated).

### Preparation of 1,3-dihydroxy-4,6-dibenzoylbenzen

Prepared as previously reported.^14^ Under N_2_, aluminium trichloride (5.734 g, 43 mmol) and dried dichloromethane (100 ml) were added to a 250 ml three-necked flask and stirred for 3 h. A solution of 1,3-dimethoxybenzene (2.5 ml, 19 mmol) and benzoyl chloride (5 ml, 43 mmol)) in dichloromethane (80 ml) was then slowly added dropwise to the stirred aluminium trichloride suspension. After the addition was complete, the reaction mixture was stirred at room temperature for 72 h. The resulting solution was then poured onto a mixture of crushed ice and concentrated hydrochloric acid (3 : 1, 150 ml). The organic layer was separated and washed with an aqueous 5% KOH solution (6 × 100 ml). The base extracts were combined, neutralized with concentrated HCl, and extracted with dichloromethane (4 × 150 ml). The dichloromethane extracts were dried (MgSO_4_) and the solvent was removed under reduced pressure. The resulting 2 g (34.3%) crude product was recrystallized from methanol to give a white solid. ^1^H NMR (400 MHz, CDCl_3_): *δ* 6.63 (s, 1H), 7.25–7.60 (m, 10H), 8.01 (s, 1H), 12.88 (s, 2H) ppm. MP = 150 °C. *ρ* = 1.312 g cm^−3^. Solubility: CH_2_Cl_2_ (ultrasonic oscillation).

### Preparation of 4,6-bis((*E*)-((4-aminophenyl)imino)(phenyl)methyl)benzene-1,3-diol (BI)

A mixture of 1,3-dihydroxy-4,6-dibenzoylphenone (1.304 g, 4.1 mmol) and *p*-phenylenediamine (0.864 g, 8 mmol) was heated by heat gun (around 180 °C) in Schlenk tube under N_2_. The mixed solid turned to dark red liquid, and then the liquid became dry and red solid was obtained. Stop heating, and the crude product was sonicated in 10 ml methanol. After filtration, the solid was dried under vacuum at 60 °C to obtain 1.76 g (88.4%) of red powder. ^1^H NMR (400 MHz, DMSO-*d*_6_): *δ* (ppm) 10.30 (s, 2H), 7.22–7.03 (m, 10H), 6.62 (s, 1H), 6.47–6.30 (m, 8H), 6.28 (s, 1H), 5.06 (s, 4H). ^13^C NMR (100 MHz, DMSO-d_6_): *δ* (ppm) 169.36, 168.89, 147.02, 142.2, 137.82, 133.80, 133.37, 129.07, 128.72, 128.70, 124.64, 114.11, 112.62, 104.69. MALDI-FTICR MS (dithranol matrix) *m*/*z* = 499.3 (M + H)^+^. Anal. Calc'd for C_32_H_26_N_4_O_2_: C (77.09%), H (5.26%), N (11.24%); found: C (77.32%), H (5.12%), N (11.26%). MP = dec at 298 °C.

### Preparation of Tp-BI-COF

A hydrothermal synthesis reactor (20 ml) is charged with Tp (17.8 mg, 0.086 mmol), BI (64 mg, 0.129 mmol), 2 ml of mesitylene, 2 ml of dioxane, 0.4 ml of 3 M aqueous acetic acid. This mixture was sonicated for 30 minutes in order to get a homogenous dispersion. The reactor was sealed off and then heated at 120 °C for 3 days. Red precipitation formed was collected by centrifugation and washed with anhydrous tetrahydrofuran and acetone. The powder collected was then dried at 180 °C under vacuum for 24 hours to give 32.44 mg dark red powder in 42.04% isolated yield. Elemental analysis: calcd: C (75.82%), H (4.24%), N (9.31%); found: C (67.68%), H (5.267%), N (9.40%). The elemental analysis is often reported to be different from the expected values as a result of incomplete combustion, as has also been found in other reports of carbon-rich porous materials.^15^ Solid-state ^13^C NMR *δ* (ppm): 186.55, 184.02, 146.44, 135.36, 129.64, 120.63, 114.68, 106.72.

### Preparation of H_3_PO_4_@Tp-BI-COF

H_3_PO_4_@Tp-BI-COF was prepared according to a reported method. ^16^*a* Under N_2_, homogeneous solution of phosphoric acid crystal (148.63 mg) dissolved in anhydrous THF (2 ml) was injected into the Tp-BI-COF sample (50 mg) in a vial (20 ml) which was preheated under vacuum at 120 °C overnight to yield a solution which was stirred at room temperature for 3 h under N_2_. The system was slowly evaporated under vacuum to remove THF at 70 °C over a period of 6 h. The vial was then kept in an oven at 70 °C under N_2_ for 12 h. The resulting powder was collected to yield H_3_PO_4_@Tp-BI-COF quantitatively.

## Results and discussion

### Characterization and properties of Tp-BI-COF

**Fig. 2:**
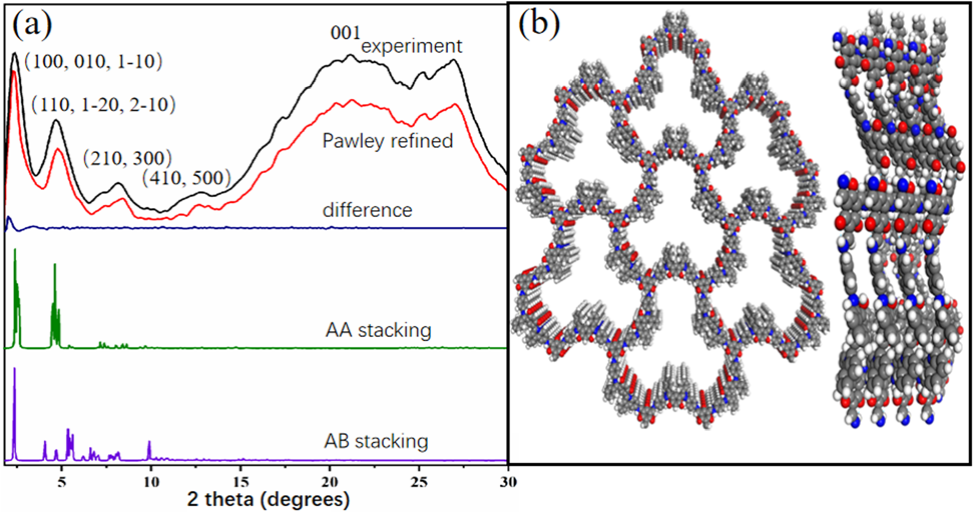
(a) PXRD patten of Tp-BI-COF. (b) Top and side view of structure of Tp-BI-COF with AA stacking with seven pores and four layers.

Powder X-ray diffraction (PXRD) pattern of Tp-BI-COF showed an intense peak at 2.1°, along with some other peaks with lower diffraction intensities, indicating long-range molecular ordering. In order to elucidate the structure of the COF, models were constructed with Materials studio software package. Two possible overlaps, eclipsed model with AA stacking and staggered model with AB stacking, were evaluated and universal force-field in the forcite module was used to minimize the geometrical energy. The experimentally obtained PXRD pattern is in good agreement with the simulated pattern of eclipsed model (Fig. 2a). As shown in Fig. 2b, according to the simulations, the layer sheet of the COF exhibits fluctuation and flexibility, which is caused by the nonplanar geometry of building block BI (Fig. S2†) and sp^3^ nitrogen node in keto-enamine moiety. To find out the unit cell parameters, Pawley refinement was performed, giving the values *a* = 38.13004 Å, *b* = 37.63950 Å, *c* = 4.25724 Å for Tp-BI-COF.

**Fig. 3:**
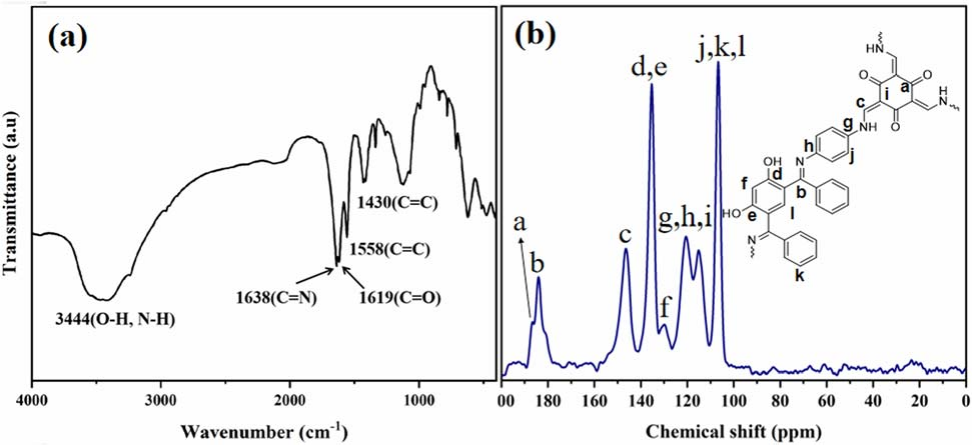
(a) IR spectrum of Tp-BI-COF. (b) ^13^C CP-MAS solid-state NMR spectrum of Tp-BI-COF.

The FT-IR spectrum of Tp-BI-COF showed a broad absorption peak appeared at 3444 cm^−1^ arising from stretching vibration of –OH (resorcinol) and –NH– present in the keto form. Peaks assigned to both carbonyl and imine groups were also appeared in IR spectrum at 1619 cm^−1^ and 1638 cm^−1^, respectively. Stretching vibration peak of conjugated C=C appeared at 1558 cm^−1^ proved the tautomerism (Fig. 3a). Thus, both enol-imine and keto-enamine moieties are present in the structure of Tp-BI-COF.

Structure of Tp-BI-COF was further demonstrated by ^13^C cross-polarization magic-angle-spinning (CP-MAS) solid-state NMR spectroscopy (Fig. 3b). The spectrum exhibited a resonance signal at 184.02 ppm, which was assigned to the C=N bond, and a shoulder peak at 186.55 ppm corresponding to the carbonyl carbon in keto-enamine moiety.

**Fig. 4:**
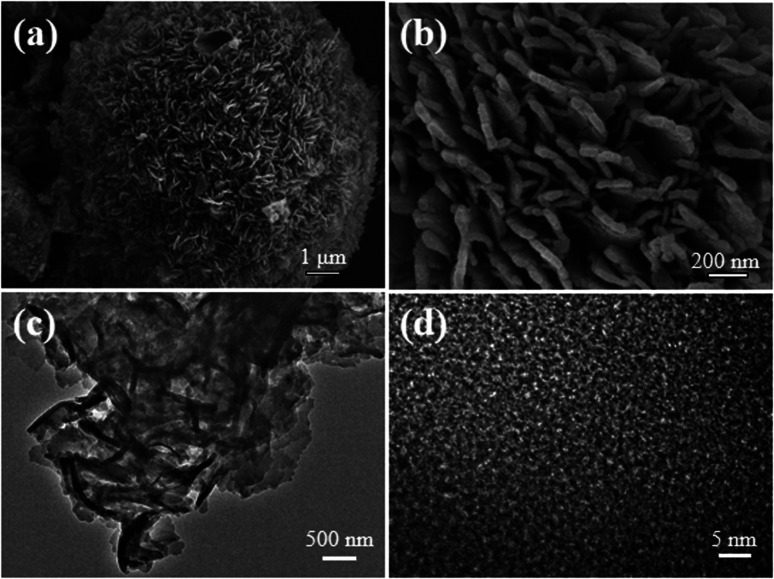
(a) (b) SEM images of Tp-BI-COF. (c) (d) HRTEM images of Tp-BI-COF.

Scanning electron microscopy (SEM) images (Fig. 4a and b and S8†) showed that the sample was mainly composed of spherical clusters of about 10–20 μm in diameter. A close view revealed that each of the clusters was stacked by an aggregation of a large number of flakes with widths in 30–50nm. Transmission electron microscopy (TEM) images also suggested the cluster of sheet-like flakes (Fig. 4c and S9†).

**Fig. 5:**
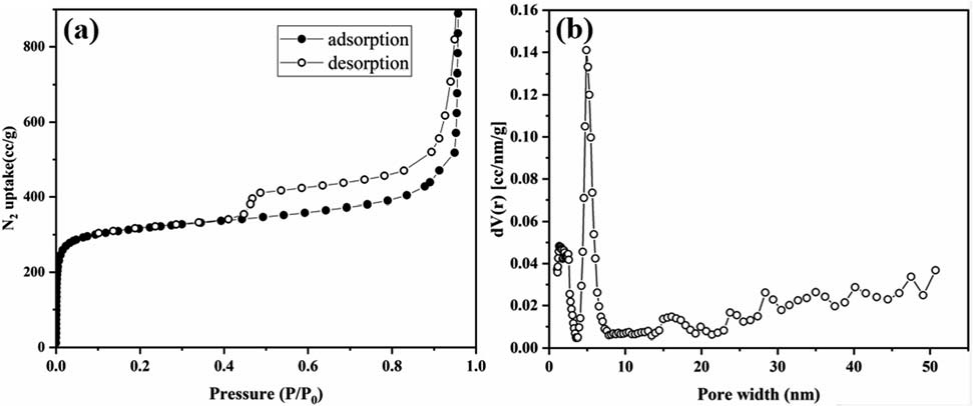
(a) N_2_ adsorption isotherm curves of Tp-BI-COF. (b) Pore size distribution of Tp-BI-COF.

Thermogravimetric analysis (TGA) was performed to determine its thermal stability and confirm the absence of guest molecules in the pores (Fig. S17†). Tp-BI-COF showed thermal stability up to 460 °C. Gradual weight losses of 30% for Tp-BI-COF was observed above 460 °C, corresponding to decomposition of the framework. The permanent porosity of Tp-BI-COF was evaluated by measuring the N_2_ adsorption isotherm at 77 K. The Brunauer–Emmett–Teller (BET) surface area of Tp-BI-COF was found to be 1018 m^2^ g^−1^. Tp-BI-COF showed a hybrid of type-I and type-IV adsorption isotherm (Fig. 5a). It featured a sharp adsorption of nitrogen into the pores at low relative pressures (*P*/*P*_0_ < 0.01), and hysteresis loop at higher relative pressure (*P*/*P*_0_ > 0.43), which is characteristic of both microporous and mesoporous nature of the material. The pore size distribution of Tp-BI-COF calculated using the nonlocal density functional theory (NLDFT) method (equilibrium model on silica), revealed the presence of 4.9 nm-sized pores (Fig. 5b), which is close to that of the lattice size (4.4 nm). Moreover, the micropores, less than 2 nm, were likely derived from interstitial spaces between flexible layers.

Chemical stability was checked by immersing Tp-BI-COF sample in THF, CH_2_Cl_2_, boiling water, aqueous HCl (9 M), H_3_PO_4_/THF solution (0.7 M). After 7 days, PXRD showed that Tp-BI-COF retained its crystallinity, as evidenced by unchanged peak positions and full width at half maximum (Fig. S19†). Nitrogen adsorption measurements indicated that the porosity of the immersed COF samples was preserved under these conditions (Fig. S20†). Stability in NaOH showed retention of PXRD peak positions and nearly unchanged surface area after treatment with 9 M NaOH for 1 day, and loss of PXRD peaks and decrease of surface area after treatment with 9 M NaOH for 2 days (Fig. S21 and S22†).

**Fig. 6:**
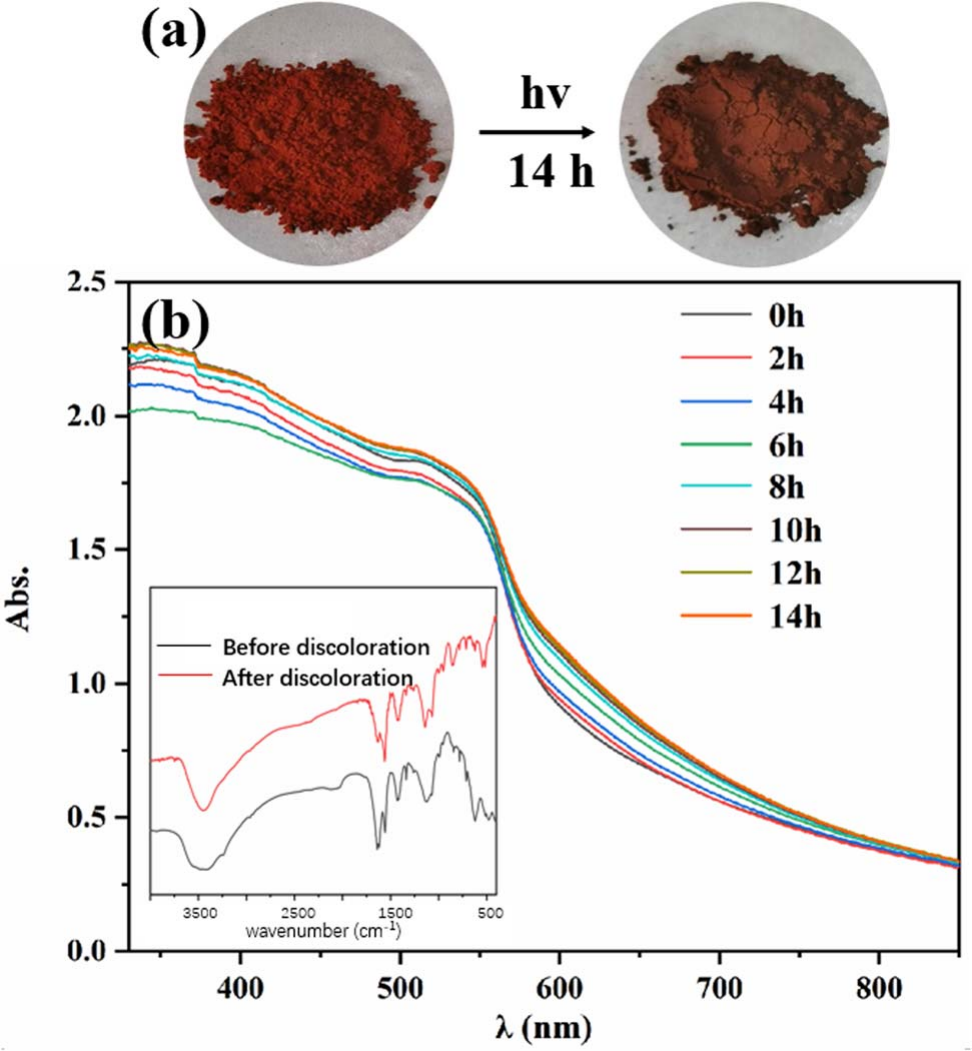
(a) Color-changing image of Tp-BI-COF before and after irradiation. (b) UV-Vis spectra of Tp-BI-COF irradiated different period of time. The insert shows FT-IR spectra before irradiation and after irradiated for 14 h.

The photochromic property of Tp-BI-COF was investigated, and a color-change, from red to dark red, was observed after being irradiated with a 300 W xenon lamp for 14 h at room temperature (Fig. 6). It was found by UV-Vis diffuse reflectance spectroscopy that after discoloration, a new absorption band appeared at 580–750 nm. The FT-IR spectra of Tp-BI-COF remained almost same before and after discoloration, except the relative intensity of peaks at 1638 cm^−1^ and 1558 cm^−1^, which were assigned to stretching vibration of C=N and C=C bonds, respectively. The PXRD pattern and BET surface area of sample which was irradiated for 14 h are similar to that of unirradiated one (Fig. S14 and S15†), indicating skeleton was retained. The mechanism of photochromism of Tp-BI-COF is still under study.

### Characterization and properties of H_3_PO_4_@Tp-BI-COF

**Fig. 7:**
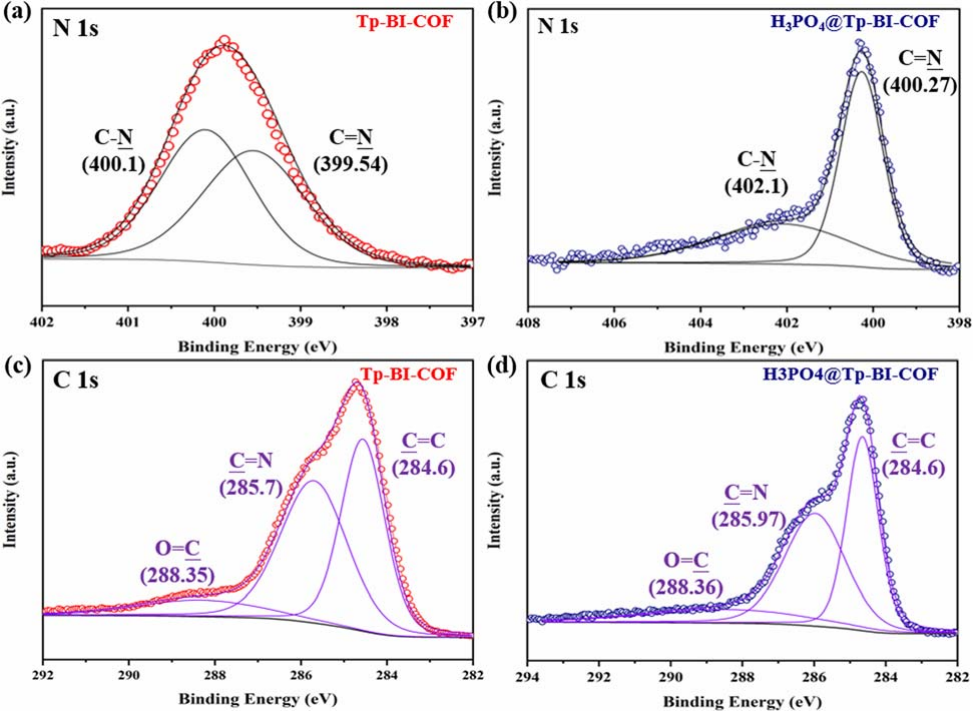
High-resolution XPS spectra of the (a) N 1s band of Tp-BI-COF, (b) N 1s band of H_3_PO_4_@Tp-BI-COF, (c) C 1s band of Tp-BI-COF, and (d) C 1s band of H_3_PO_4_@Tp-BI-COF.

Anhydrous proton conduction, which enables high temperature proton transport based on pure H_3_PO_4_, requires high stability of porous material and proton network.^16^ Because one macrocycle of Tp-BI-COF contains twelve inward N_in_ from C=N and C–N units, which enables anchoring and stabilizing H_3_PO_4_ network on pore walls in aligned channels, the stable Tp-BI-COF was expected to provide freeway for proton flow. In order to loaded pure H_3_PO_4_ crystal into the channels of framework, H_3_PO_4_@Tp-BI-COF was prepared *via* vacuum impregnation method.^16*a*^ The generated H_3_PO_4_@Tp-BI-COF sample showed negligible porosity (Fig. S12†), indicating that the pores of Tp-BI-COF were filled with H_3_PO_4_. From the density of pure H_3_PO_4_ (1.834 g cm^−3^) and the pore volume of Tp-BI-COF (1.62 cm^3^ g^−1^), the maximum H_3_PO_4_ loading was calculated as 74.8 wt%. New peaks at 969 and 510 cm^−1^ present in FT-IR spectrum of the composite, which were attributed to P=O bond, demonstrated the formation of H_3_PO_4_@Tp-BI-COF (Fig. S7†). The EDS mapping indicated the homogeneously distribution of P element throughout H_3_PO_4_@Tp-BI-COF (Fig. S25†). The vibrational band of the C=N bond shifted to 1688 cm^−1^, indicating the N atom of the C=N bond interacted with H_3_PO_4_.^16^ Interaction between H_3_PO_4_ and Tp-BI-COF was studied by X-ray photoelectron spectroscopy (XPS) (Fig. 7 and S27†). The binding energies of the C 1s spectrum of Tp-BI-COF were observed at 284.6, 285.7 and 288.35 eV, which were attributed to the C=C, C=N and C=O bonds in the COF. The shift of the second peak from 285.7 to 285.97 eV for H_3_PO_4_@Tp-BI-COF was attributed to the ionization of the imine C=N bond to the iminium cation (C=NH^+^).^17^ Two peaks at 399.54 and 400.1 eV appeared in high-resolution N 1s spectrum of Tp-BI-COF, which were assigned to the N atoms of the C–N bond and imine C=N bond, shifted to 400.27 eV and 402.1 eV for H_3_PO_4_@Tp-BI-COF, respectively, reflecting N atoms in both C–N and C=N bonds interacted with H_3_PO_4_. In the P 2p XPS spectrum of H_3_PO_4_@Tp-BI-COF, two peaks were observed at 134.2 and 135.0 eV, which were assigned to the phosphorus atoms of H_2_PO_4_^−^ and H_3_PO_4_, respectively. Based on FT-IR and XPS data, we suggest both N atoms in C–N and C=N bond on the pore walls are protonated by H_3_PO_4_ (Fig. S16†), and H_3_PO_4_ network in the channel is restricted and stabilized through hydrogen bond.

**Fig. 8:**
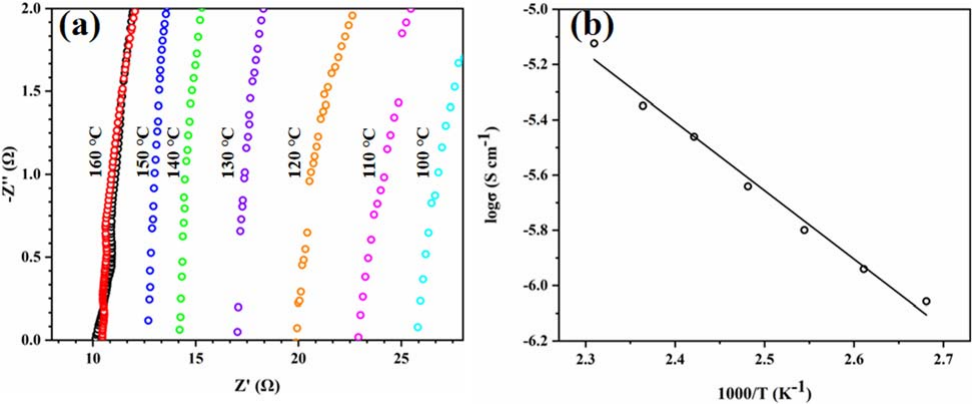
(a) Nyquist plots of H_3_PO_4_@Tp-BI-COF at 100 °C (light blue), 110 °C (pink), 120 °C (orange), 130 °C (purple), 140 °C (green), 150 °C (blue)**,** 160 °C (red) and 160 °C after a 120 h run (black). (b) Arrhenius plots of H_3_PO_4_@Tp-BI-COF.

Tp-BI-COF is an insulator (conductivity = 4.8 × 10^−9^ S cm^−1^ at 160 °C), which makes it suitable for proton-conducting study. Proton conductivity of pellet of H_3_PO_4_@Tp-BI-COF, which was prepared at a pressure of 200 kN under nitrogen for 30 min, was measured by alternating-current impedance spectroscopy under anhydrous conditions under nitrogen at varying temperatures from 100 to 160 °C. The proton conductivities were calculated to be 2.34 × 10^−3^, 2.63 × 10^−3^, 3.03 × 10^−3^, 3.55 × 10^−3^, 4.25 × 10^−3^, 4.75 × 10^−3^, and 5.95 × 10^−3^ S cm^−1^ at 100 °C, 110 °C, 120 °C, 130 °C, 140 °C, 150 °C, and 160 °C, respectively (Fig. 8a). Under anhydrous conditions, the proton flow is on the same order of magnitude as other similar COFs (Table S3†). We tested the long-term stability of H_3_PO_4_@Tp-BI-COF at 160 °C for 120 h and observed that the proton conductivity almost remained at the same value as its initial value (Fig. S28†), and PXRD of sample after long-term test retained unchanged as well (Fig. S23†). As shown in Fig. 8b, an Arrhenius-type linear curve is derived. According to the slope of the curve, the activation energy was estimated to be 0.21 eV. These results suggest that Tp-BI-COF can give a way to design well-defined porous material for anhydrous proton conduction.

## Conclusions

In summary, a crystalline COF with both ketimine-type enol-imine and keto-enamine tautomeric linkages is introduced for the first time. The porous framework is thermally and chemically stable. The material shows photochromic property and stable anhydrous proton conduction over a wide temperature range above 100 °C. This work provides further aid to the fields of photochromism and anhydrous proton conduction.

## Conflicts of interest

There are no conflicts to declare.

## Supplementary Material

RA-013-D3RA02251J-s001
