# Supplementary material for: Construction of highly-stable covalent organic framework with combined enol-imine and keto-enamine linkages
Source: RSC Adv. 2023 May 15;13(22):14776–81. doi: 10.1039/d3ra02251j (PMC10184012; doi:10.1039/d3ra02251j)
Supplement: RA-013-D3RA02251J-s001 [file RA-013-D3RA02251J-s001.pdf]

Electronic Supplementary Material (ESI) for RSC Advances.
This journal is © The Royal Society of Chemistry 2023

Construction of High-Stable Covalent Organic Framework with Combined Enol-imine and Keto-enamine Tautomeric Linkages

Jian Jiang, Kaiyu He, Xue Cai, Hong Yu, Minghui Zuo, Guan Yun, Tao Yun, Yue Ma, Zitong Zhang, Yunling Liu, Zhenlu Wang*

Support Information

Section		Page No
S-1	General Information	S2
S-2	Synthetic Procedures	S5
S-3	Structure Modeling and Atomic Coordinates of COFs	S9
S-4	FT-IR Spectra	S17
S-5	Morphological study of Tp-BI-COF	S19
S-6	Optimization of Tp-BI-COF Synthesis Scheme	S20
S-7	Gas Adsorption Studies	S22
S-8	Photochromic properties of Tp-BI-COF	S23
S-9	Preparation of H_3PO_4@Tp-BI-COF	S25
S-10	Stability study of Tp-BI-COF and H_3PO_4@Tp-BI-COF	S26
S-11	PXRD study of Tp-BI-COF and H_3PO_4@Tp-BI-COF	S29
S-12	Energy dispersive X-ray spectroscopy	S30
S-13	High-resolution XPS spectra	S31
S-14	Nyquist plots of H_3PO_4@Tp-BI-COF	S32
S-15	Current status of anhydrous proton conductors	S33
S-16	References	S34

Section S-1: General Information

A. Materials

Unless stated otherwise all reagents were purchased from commercial source and used without purification. Benzoyl chloride, p-phenylenediamine and pure H_3PO_4 crystal were purchased from Shanghai Aladdin Biochemical Technology Co., Ltd. Trifluoroacetic acid, 2,4,6-trimethylbenzaldehyde, mesitylene and ultra-dry 1,4-dioxane were purchased from Beijing InnoChem Science & Technology Co., Ltd. Dichloromethane, methanol and tetrahydrofuran were purchased from Zhongtian Fine Chemical Co., Ltd. Anhydrous aluminum trichloride, hydrochloric acid, glacial acetic acid and N,N-dimethylformamide were purchased from Tianjin Damao Chemical Reagent Factory. Potassium hydroxide, sodium hydroxide, anhydrous magnesium sulfate and potassium bromide from Tianjin Bodi Chemical Co., Ltd. Hexamethylenetetramine from China Pharmaceutical (Group) Shanghai Chemical Reagent Company and phloroglucinol from Tianjin BASF Chemical Co., Ltd. 1,3,5-triformylphloroglucinol[1] and 1,3-dihydroxy-4,6-dibenzoylbenzen[2] **(Tp)** was synthesized according to literature methods. P-phenylenediamine was purified by sublimation before use.

B. Instruments and methods

X-ray powder diffraction (PXRD) patterns were recorded on a Rigaku Ultima IV-185 diffractometer using Cu Kα radiation (λ = 1.54056 Å) over the 2θ range of 1.8°-30° and on a SAXSess mc2 diffractometer using Cu Kα radiation (λ = 1.54056 Å) over the 2θ range of 0.5°-5°. The Shimadzu UV-2600 UV-Vis spectrophotometer, equipped with integration sphere ISR-2200, was used to measure the UV-Vis diffuse reflectance spectrum of solid powders at room temperature. FT-IR spectra were measured on a PerkinElmer Frontier infrared spectrometer FTIR-650 ranging from 4000 to 400 cm^{-1}. The thermogravimetric analysis was performed on a Netzsch TG 209 F1 Libra thermogravimetric analyzer with a ramp rate of 10 K/min and a temperature of 35 to 800 °C under the atmosphere of nitrogen. Elemental analysis (C, H, O and N) was

performed on a PerkinElmer 2400chn elemental analyzer. SEM images were obtained using a JSM-6480LV at 5.0 kV. FEI (Jeol FEG 2100F) high resolution transmission electron microscope (HRTEM) equipped with field emission source operating at 300 kV was used to record TEM images. The nitrogen adsorption and desorption isotherms were measured at 77 K using an Autosorb-iQ (Quantachrome) surface area size analyzer. Before measurement, the samples were degassed in vacuum at 100 °C for 10 h. The Brunauer-Emmett-Teller (BET) method was utilized to calculate the specific surface area. ^{1}H and ^{13}CNMR spectrum were measured on a Bruker Fourier 400 MHz spectrometer. Solid state cross polarization magic angle spinning (CP/MAS) NMR was taken on an AVIII 500 MHz solid-state NMR spectrometer. Mass spectra were obtained on the SolariX XR-15T FTMS Mass Spectrometry Facility. X-ray photoelectron spectroscopy (XPS) was measured on the Thermo ESCALAB 250XI instrument and the C 1s line at 284.8 eV was used as the binding energy reference.

Impedance Measurements. The pellet was sandwiched between two stainless steel electrodes under nitrogen. The proton conductivity was characterized by alternating current impedance spectroscopy using a CHI 600E instrument over frequency range 1 Hz to 1 MHz with input amplitude voltage of 100 mV under nitrogen. To estimate the activation energy (*Ea*) of the solid electrolyte, the conductivity was measured at various temperatures from 100 to 160 °C. The conductivity was calculated using the equation of $\sigma = L/SRs$ where σ is the conductivity (S cm^{-1}), and S is the electrode area (cm^2), L is the sample pellets thickness (cm), and Rs is the bulk resistance of the sample (Ω). The activation energy (*Ea*) for the material conductivity was estimated from the equation of $\sigma = \sigma_0 e^{-Ea/k_B T}$, where σ_0 is the pre-exponential factor, k_B is the Boltzmann constant, and T is the sample temperature.

Calculation of maximum loading content of H_3PO_4.

The maximum loading content of H_3PO_4 in **H_3PO_4@Tp-BI-COF** is calculated as formula below:

$$\text{Maximum loading content} = \frac{d \cdot V}{d \cdot V + 1}$$

d: density of H_3PO_4 (g cm^{-3})

V: pore volume of COF (cm^3 g^{-1})

Section S-2: Synthetic procedures

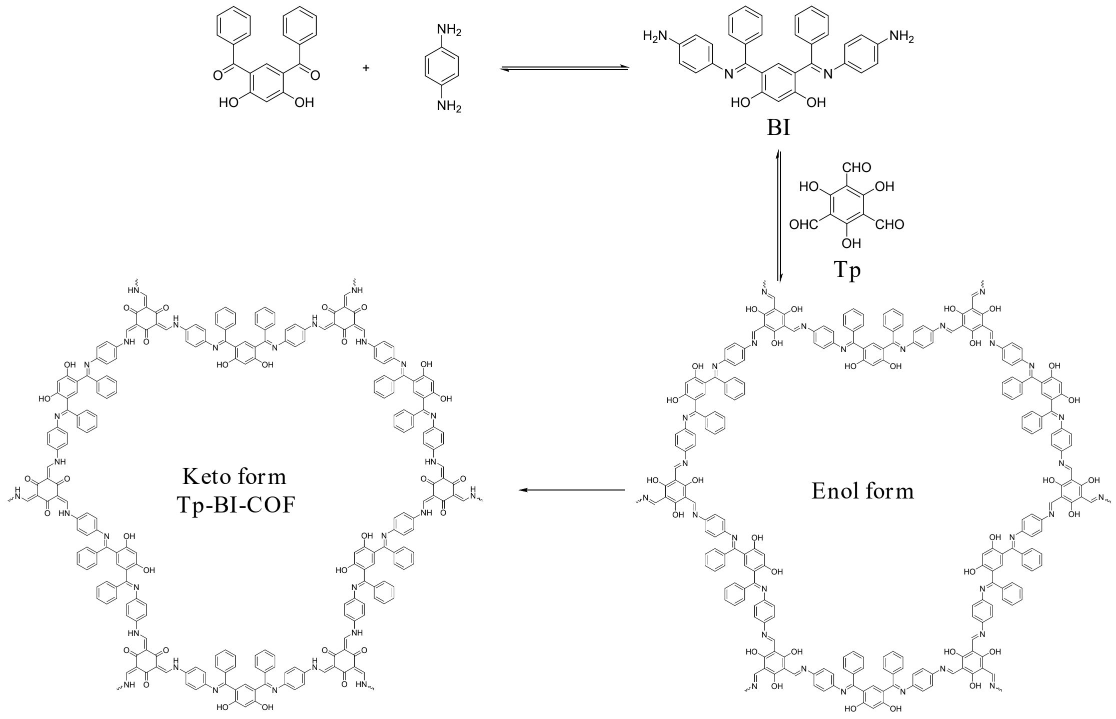

Figure S1: Schematic representation of the synthesis of **Tp-BI-COF**.

Synthesis of 4,6-bis((E)-((4-aminophenyl)imino)(phenyl)methyl)benzene-1,3-diol (BI): A mixture of 1,3-Dihydroxy-4,6-dibenzoylphenone (1.304 g, 4.1 mmol) and p-phenylenediamine (0.864 g, 8 mmol) was heated by heat gun (around 180 °C) in Schlenk tube under N_2. The mixed solid turned to dark red liquid, and then the liquid became dry and red solid was obtained. Stop heating, and the crude product was sonicated in 10 ml methanol. After filtration, the solid was dried under vacuum at 60 °C to obtain 1.76 g (88.4 %) of red powder.

Data of 4,6-bis((E)-((4-aminophenyl)imino)(phenyl)methyl)benzene-1,3-diol (BI): ^{1}H NMR (400 MHz, DMSO-d_6): δ (ppm) 10.30 (s, 2H), 7.22-7.03 (m, 10H), 6.62 (s, 1H), 6.47-6.30 (m, 8H), 6.28 (s, 1H), 5.06 (s, 4H). ^{13}C NMR (100 MHz, DMSO-d_6): δ (ppm) 169.36, 168.89, 147.02, 142.2, 137.82, 133.80, 133.37, 129.07, 128.72, 128.70, 124.64, 114.11, 112.62, 104.69. MALDI-FTICR MS (dithranol matrix) *m/z* = 499.3

$(M+H)^+$. Anal. Calc'd for $C_{32}H_{26}N_4O_2$: C (77.09 %), H (5.26 %), N (11.24 %); Found: C (77.32 %), H (5.12%), N (11.26 %). MP = dec. at 298 °C.

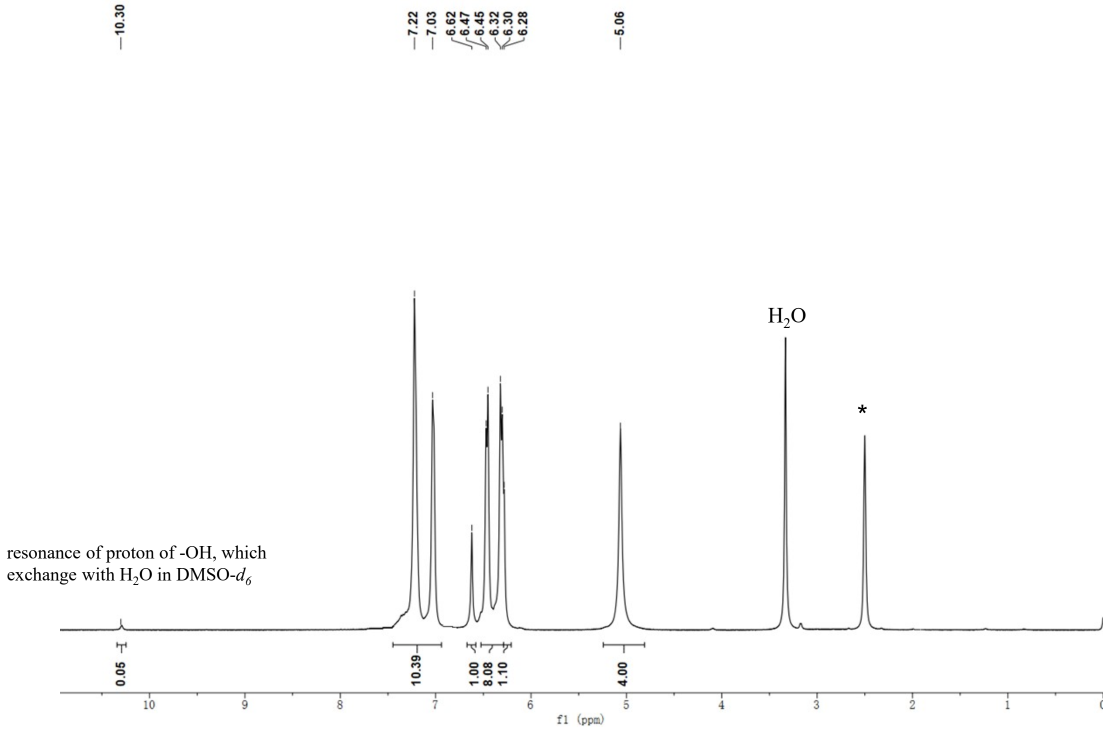

^{1}H NMR spectrum of **BI** in DMSO-d_6.

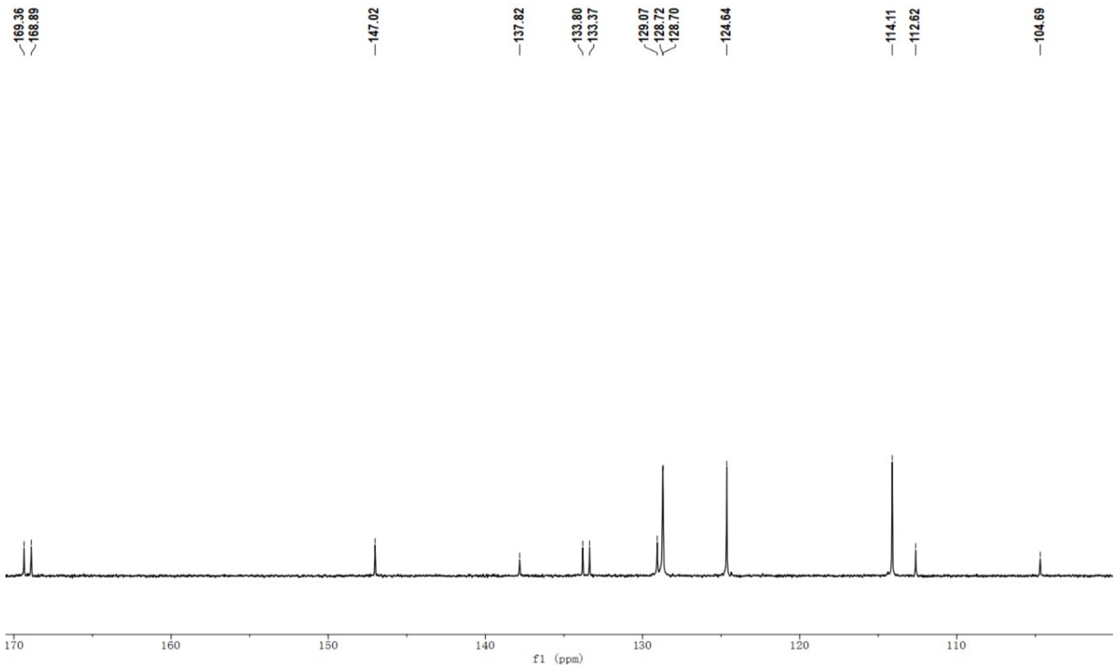

^{13}C NMR spectrum of **BI** in DMSO-d_6.

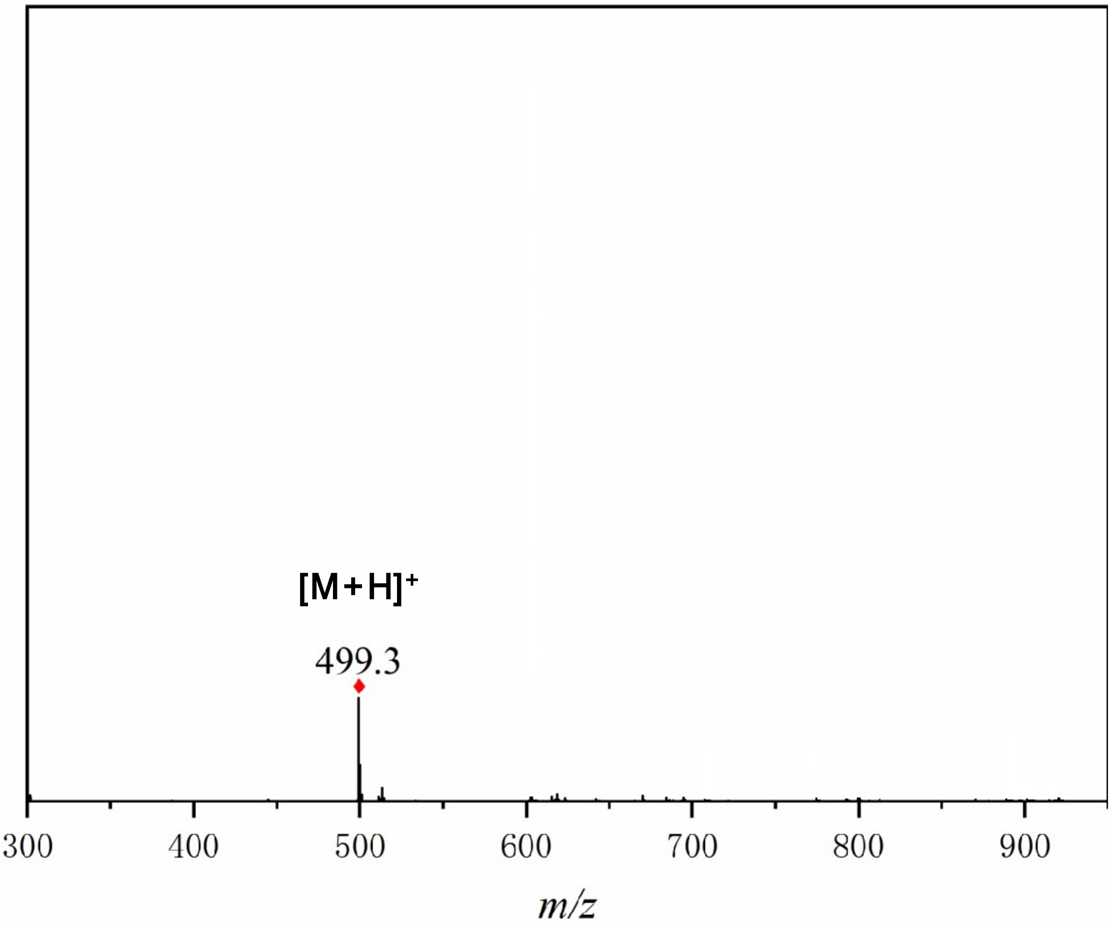

MALDI-FTICR MS spectrum of **BI**.

Synthesis of Tp-BI-COF: A hydrothermal synthesis reactor (20 ml) is charged with **Tp** (17.8 mg, 0.086 mmol), **BI** (64 mg, 0.129 mmol), 2 mL of mesitylene, 2 ml of dioxane, 0.4 ml of 3 M aqueous acetic acid. This mixture was sonicated for 30 minutes in order to get a homogenous dispersion. The reactor was sealed off and then heated at 120 °C for 3 days. Red precipitation formed was collected by centrifugation and washed with anhydrous tetrahydrofuran and acetone. The powder collected was then dried at 180 °C under vacuum for 24 hours to give 32.44 mg dark red powder in 42.04 % isolated yield.

Data for Tp-BI-COF: Elemental analysis: Calcd: C (75.82 %), H (4.24 %), N (9.31 %); Found: C (67.68 %), H (5.267 %), N (9.40 %). The elemental analysis is often reported to be different from the expected values as a result of incomplete combustion, as has also been found in other reports of carbon-rich porous materials.[3] Solid-state ^{13}C

NMR δ (ppm): 186.55, 184.02, 146.44, 135.36, 129.64, 120.63, 114.68, 106.72.

Preparation of H_3PO_4@Tp-BI-COF: H_3PO_4@Tp-BI-COF was prepared according to a reported method.[4] Homogeneous solution of phosphoric acid crystal (148.63 mg) dissolved in anhydrous THF (2 mL) was injected into the **Tp-BI-COF** sample (50 mg) in a vial (20 mL) which was preheated under vacuum at 120 °C overnight to yield a solution which was stirred at room temperature for 3 h under N_2. The system was slowly evaporated under vacuum to remove THF at 70 °C over a period of 6 h. The vial was then kept in an oven at 70 °C under N_2 for 12 h. The resulting powder was collected to yield **H_3PO_4@Tp-BI-COF** quantitatively.

Stability test and COF sample recovery methods.

Stability test: The COF samples (100 mg) were dispersed in different solvents including water (100 °C), THF, CH_2Cl_2, aqueous HCl (9 M), and neat H_3PO_4 (9 M, a THF solution of H_3PO_4), and stirred for one week. Stability in base circumstance was tested in aqueous NaOH solution by dispersing COF sample (100 mg) into aqueous NaOH (9 M) and stirring for one or two days.

Sample recovery methods: The COF samples in boiling water, THF, and CH_2Cl_2 were collected by filtration and dried at 120 °C under vacuum for 12 h. The COF samples in THF solution of H_3PO_4 was washed with CH_3OH (2 × 10 mL) and water (10 × 10 mL), neutralized with triethylamine/acetone solution (5 %, 1 × 10 mL), washed again with water (5 × 10 mL) and dried under vacuum at 120 °C for 12 h. The COF sample in the aqueous HCl solution was washed with water (5 × 10 mL), neutralized with triethylamine/acetone solution (5 %, 1 × 10 mL), washed again with water (5 × 10 mL), and dried under vacuum at 120 °C for 12 h. COF sample in aqueous NaOH solution was washed with water (20 × 10 ml) and dried under vacuum at 120 °C for 12 h. These samples were then used for PXRD and porosity measurements.

Section S-3: Structure Modeling and Atomic Coordinates of COF

The geometry of compound **BI** was optimized with Materials Studio suite of programs to determine the conformations. Simulation was started from likely conformation and minimized using universal force-field in the forcite module.

The **Tp-BI-COF** models were simulated using Materials Studio suite of programs. Firstly, the eclipsed model was built and the symmetry of lattice was degraded to *P1*. Then the lattice and geometry model were optimized using universal force-field in the forcite module. The staggered arrangement for **Tp-BI-COF** was also examined by offsetting the alternating stacked units from the eclipsed model.

Pawley refinement was carried out using Reflex module, a software package for crystal determination from PXRD pattern. Unit cell dimension was set to the theoretical parameters. The Pawley refinement was performed to optimize the lattice parameters iteratively until the Rwp value converges and overly of the observed with refined profiles show good agreement.

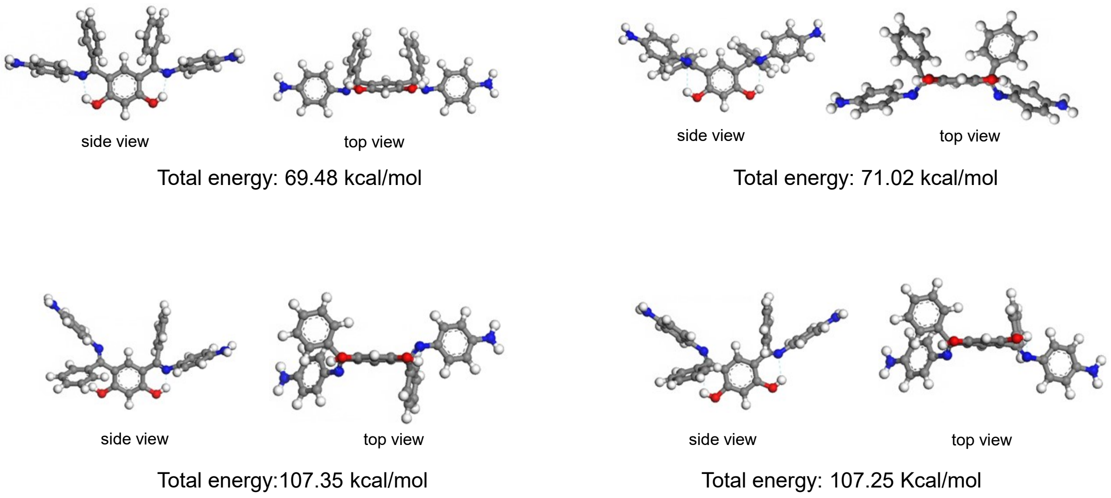

Figure S2: Energy-minimized structure of **BI**. Atom key: N (blue), C (gray), H(white), O (red). From the simulation, several conformations with similar energy were obtained. There may well be additional conformation, but these are representative. What they clearly show is that the compound appears flexible and does not have a flat conformation.

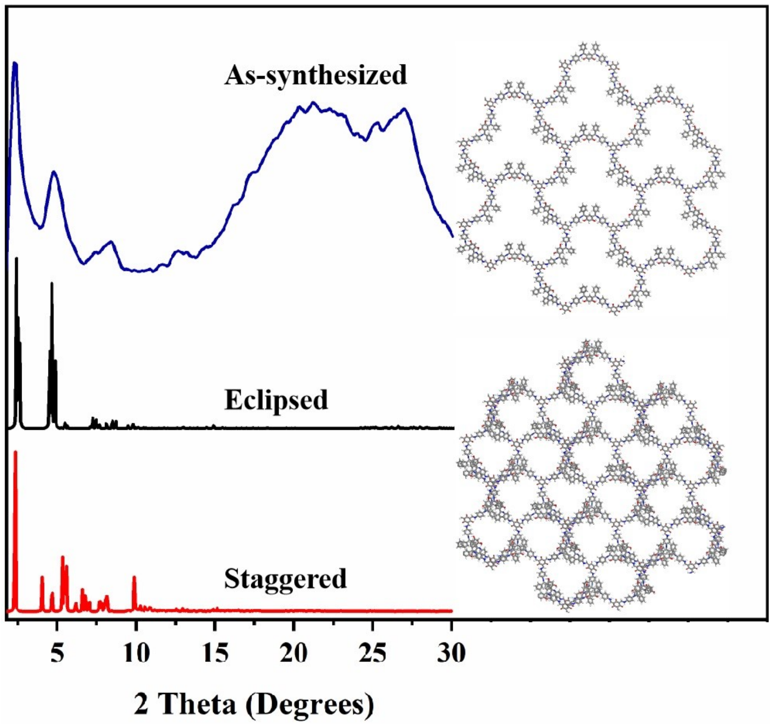

Figure S3: PXRD patterns of **Tp-BI-COF:** experimental pattern (blue), simulated patterns with the eclipsed (black) and staggered (red) stacking models.

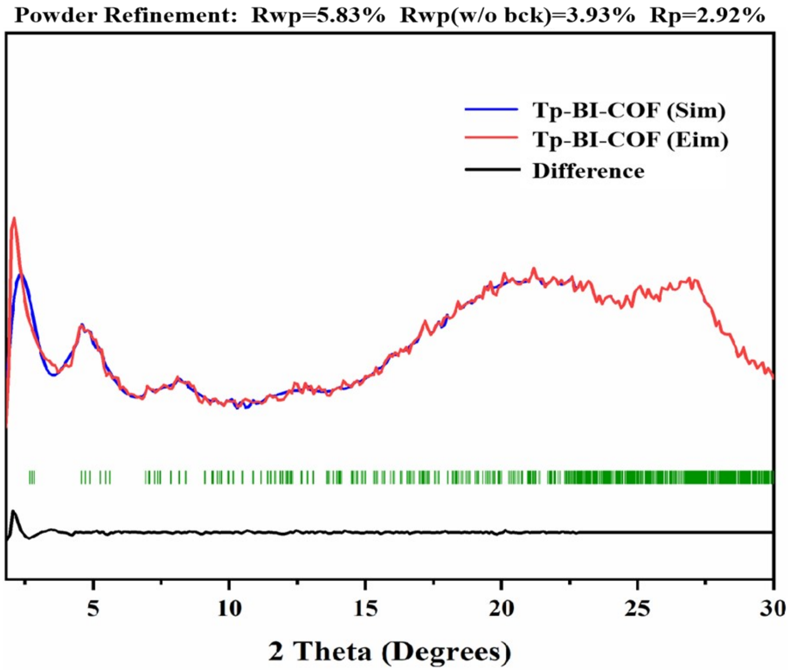

Figure S4: PXRD profiles of **Tp-BI-COF** experimental observed (red), Pawley refinement (blue) and their difference (black).

Change in unit cell parameters of Tp-BI-COF	
**Before**	**After refinement**
**Space group: P1(C1-1)**	**Space group: P1(C1-1)**
**a = 39.0585 Å, b = 37.5546 Å, c = 3.96141 Å**	**a = 38.13004 Å, b = 37.63950 Å, c = 4.25724 Å**
**α = 103.110, β = 92.6854, γ = 118.231**	**α = 103.097, β = 92.2914, γ = 118.257**

Table S1. Fractional atomic coordinates for the unit cell of Tp-BI-COF

Tp-BI-COF			
Triclinic P1(C1-1)<br>a = 38.13004 Å, b = 37.63950 Å, c = 4.25724 Å			
Atom	x/a	y/b	z/c
H	18.528	15.454	-1.392
H	14.431	10.613	-0.12
C	22.345	23.066	2.135
C	21.586	24.23	2.168
C	20.492	24.377	1.315
C	20.174	23.345	0.43
C	20.956	22.197	0.39
C	22.078	22.021	1.229
O	24.167	18.253	1.968
C	24.833	19.454	1.98
C	26.16	19.386	2.363
C	26.957	20.512	2.455
C	26.42	21.778	2.162
C	25.086	21.809	1.745
C	24.246	20.694	1.65
C	22.856	20.782	1.184
N	22.271	19.73	0.697
C	21.654	16.382	-0.803
C	22.312	17.504	-0.309
C	21.608	18.614	0.172
C	20.211	18.58	0.107

C	19.537	17.479	-0.404
C	20.246	16.345	-0.847
N	20.141	9.068	0.829
N	19.511	15.237	-1.318
O	16.233	9.928	0.168
C	18.836	9.37	0.547
O	20.623	11.557	0.037
C	19.867	13.99	-0.87
O	17.054	14.308	-1.24
C	17.006	10.841	-0.076
C	18.448	10.61	0.115
C	19.423	11.705	-0.144
C	18.94	13.01	-0.631
C	17.486	13.24	-0.832
C	16.526	12.166	-0.528
C	15.195	12.452	-0.658
N	14.194	11.562	-0.366
C	10.883	11.464	1.35
C	12.04	11.099	0.627
C	13.046	12.02	0.34
C	12.901	13.352	0.793
C	11.779	13.735	1.523
C	10.809	12.803	1.797
C	7.67	10.087	2.824
C	7.742	8.764	2.409
C	8.812	8.33	1.626
C	9.809	9.211	1.272
C	9.776	10.567	1.673
C	2.045	11.582	3.303
C	1.583	10.279	3.157
C	2.377	9.206	3.565
C	3.631	9.456	4.124
C	4.082	10.763	4.264
C	3.312	11.87	3.851
N	9.649	12.974	2.488
C	8.626	11.024	2.456
C	8.58	12.424	2.89
O	9.186	14.784	4.483
O	4.723	15.441	5.695
C	6.203	12.835	3.568
C	7.557	13.163	3.62
C	7.902	14.306	4.368

C	6.928	15.01	5.051
C	5.592	14.661	4.975
C	5.19	13.558	4.199
C	3.775	13.248	4.002
N	2.939	14.235	3.876
C	-0.087	16.333	3.29
C	0.716	15.219	3.515
C	2.09	15.345	3.747
C	2.616	16.638	3.802
C	1.832	17.763	3.578
C	0.46	17.633	3.281
C	21.671	-0.856	1.267
C	22.417	0.305	1.524
C	21.898	1.539	1.936
C	20.505	1.591	2.114
C	19.739	0.458	1.925
C	20.289	-0.746	1.531
O	19.821	2.708	2.516
C	22.726	2.736	2.138
N	22.21	3.914	1.932
C	21.663	5.191	1.712
C	22.389	6.357	1.982
C	21.876	7.618	1.701
C	20.589	7.769	1.146
C	19.839	6.6	0.909
C	20.374	5.348	1.19
C	24.137	2.653	2.523
C	25.01	3.745	2.33
C	26.355	3.692	2.682
C	26.886	2.528	3.238
C	26.046	1.438	3.467
C	24.699	1.515	3.136
C	4.488	30.948	0.812
C	5.853	30.843	0.279
N	3.804	29.842	0.9
C	6.549	29.614	0.3
C	7.851	29.484	-0.17
C	8.518	30.59	-0.696
C	7.843	31.807	-0.787
C	6.534	31.914	-0.332
C	3.113	28.62	0.991
C	1.789	28.485	0.552

C	1.102	27.278	0.644
C	1.733	26.131	1.165
C	3.074	26.256	1.58
C	3.735	27.475	1.501
N	1.115	24.871	1.309
C	-0.218	24.63	1.103
C	-0.785	23.408	1.352
C	-2.228	23.256	1.102
C	-2.898	21.957	1.377
C	-2.121	20.819	1.903
C	-0.665	20.976	2.146
C	0.004	22.258	1.856
O	-2.835	24.22	0.661
C	-4.238	21.777	1.168
O	-2.695	19.766	2.135
C	0.11	19.964	2.642
O	1.208	22.34	2.043
N	-0.382	18.724	2.97
N	-5.044	22.781	0.693
C	-6.417	22.77	1.013
C	-7.022	23.968	1.44
C	-8.371	24.001	1.769
C	-9.181	22.866	1.652
C	-8.58	21.684	1.207
C	-7.224	21.621	0.902
N	-10.552	22.919	1.945
C	-11.82	22.972	2.217
C	-12.417	24.252	2.591
C	-11.794	25.475	2.267
C	-12.34	26.699	2.636
C	-13.542	26.745	3.344
C	-14.174	25.551	3.697
C	-13.609	24.333	3.339
H	20.478	3.397	2.604
H	30.968	47.465	3.799
H	25.129	48.199	5.53
H	23.005	57.239	1.659
H	19.936	51.579	2.816
H	44.539	51.557	1.771
O	10.468	53.379	2.863
H	10.863	54.244	2.956
O	40.674	31.319	1.442

H	41.2	30.526	1.38
H	16.615	56.81	0.635
H	42.136	42.873	0.839
H	23.132	22.971	2.89
H	21.829	25.022	2.891
H	19.883	25.29	1.343
H	19.318	23.45	-0.252
H	20.679	21.428	-0.345
H	26.592	18.408	2.613
H	24.694	22.781	1.415
H	22.241	15.531	-1.185
H	23.413	17.518	-0.3
H	19.619	19.43	0.472
H	18.434	17.473	-0.433
H	18.105	8.575	0.715
H	20.933	13.817	-0.704
H	14.861	13.435	-0.997
H	12.177	10.07	0.259
H	13.682	14.104	0.604
H	11.67	14.772	1.869
H	6.834	10.337	3.485
H	6.965	8.051	2.719
H	8.867	7.278	1.307
H	10.644	8.819	0.676
H	1.391	12.389	2.948
H	0.594	10.094	2.713
H	2.015	8.174	3.456
H	4.257	8.62	4.467
H	5.062	10.896	4.734
H	5.899	12.014	2.909
H	7.221	15.882	5.651
H	-1.164	16.202	3.09
H	0.245	14.228	3.478
H	3.692	16.786	3.988
H	2.319	18.748	3.641
H	23.503	0.259	1.397
H	18.656	0.515	2.102
H	23.395	6.301	2.417
H	22.472	8.518	1.924
H	18.837	6.653	0.457
H	19.76	4.464	0.959
H	24.658	4.666	1.85

H	27.003	4.561	2.499
H	27.949	2.477	3.512
H	26.443	0.531	3.947
H	24.075	0.659	3.407
H	6.091	28.715	0.729
H	8.36	28.511	-0.11
H	9.547	30.496	-1.067
H	8.335	32.672	-1.255
H	6.031	32.868	-0.509
H	1.261	29.339	0.103
H	0.067	27.238	0.273
H	3.595	25.378	1.997
H	4.767	27.52	1.872
H	-0.817	25.465	0.735
H	-4.725	20.824	1.39
H	1.179	20.124	2.798
H	-6.408	24.879	1.537
H	-8.791	24.954	2.117
H	-9.195	20.781	1.071
H	-6.791	20.678	0.533
H	-10.867	25.493	1.68
H	-11.834	27.632	2.35
H	-13.98	27.71	3.633
H	-15.103	25.572	4.285
H	-14.106	23.427	3.706

Section S-4: FT-IR Spectra

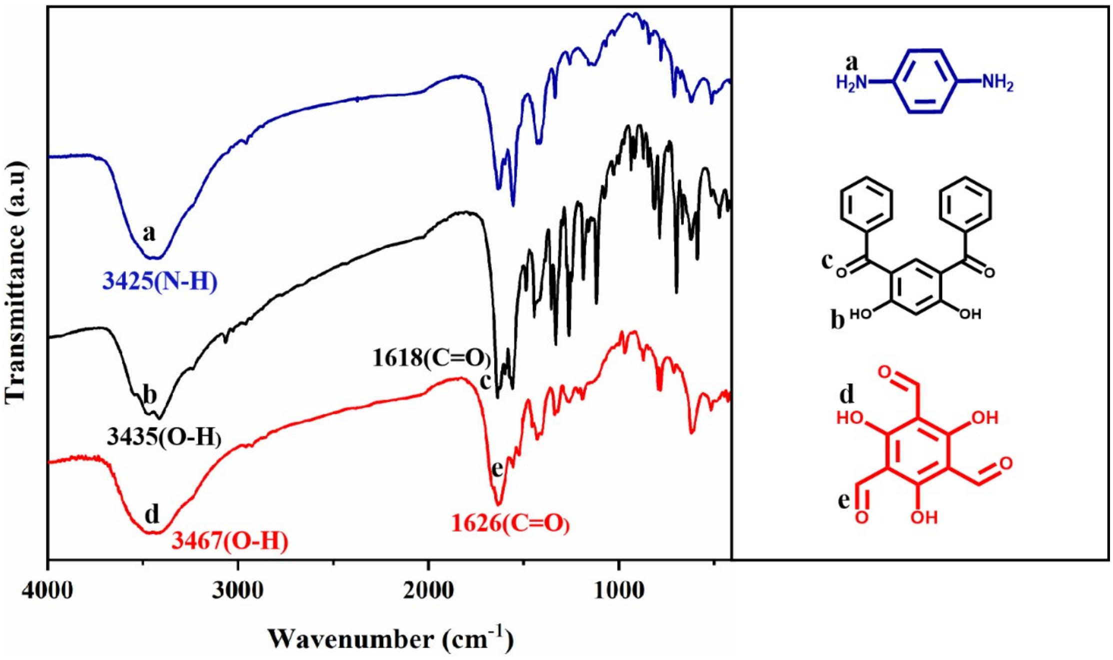

Figure S5: FT-IR spectra of p-phenylenediamine (blue), 1,3-dihydroxy-4,6-dibenzoylbenzen (black), and triformylphloroglucinol (red).

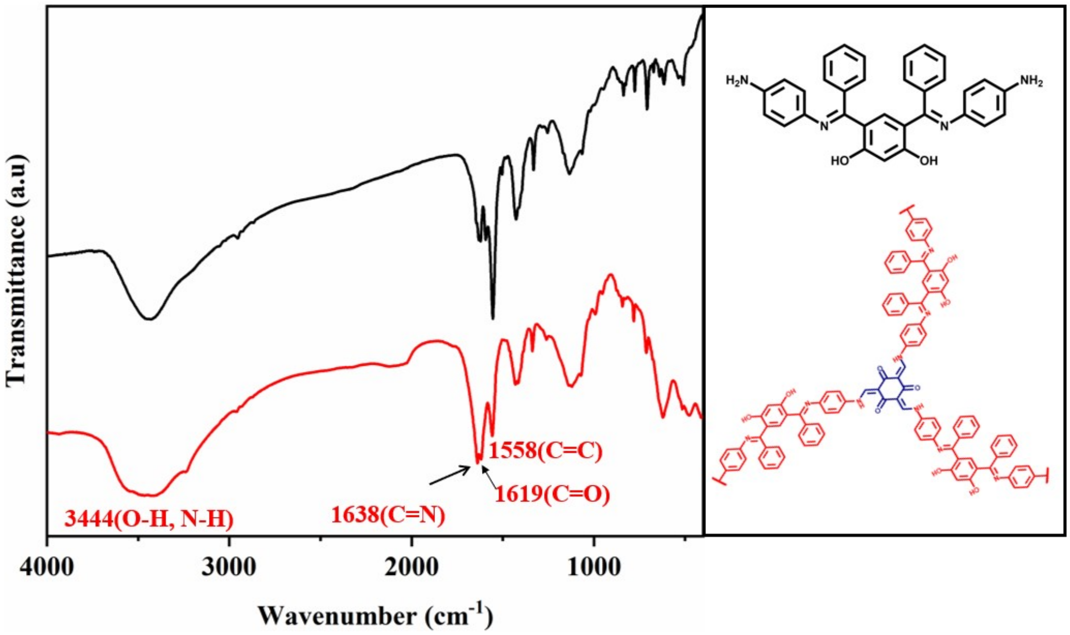

Figure S6: FT-IR spectra of **BI** (black) and **Tp-BI-COF** (red).

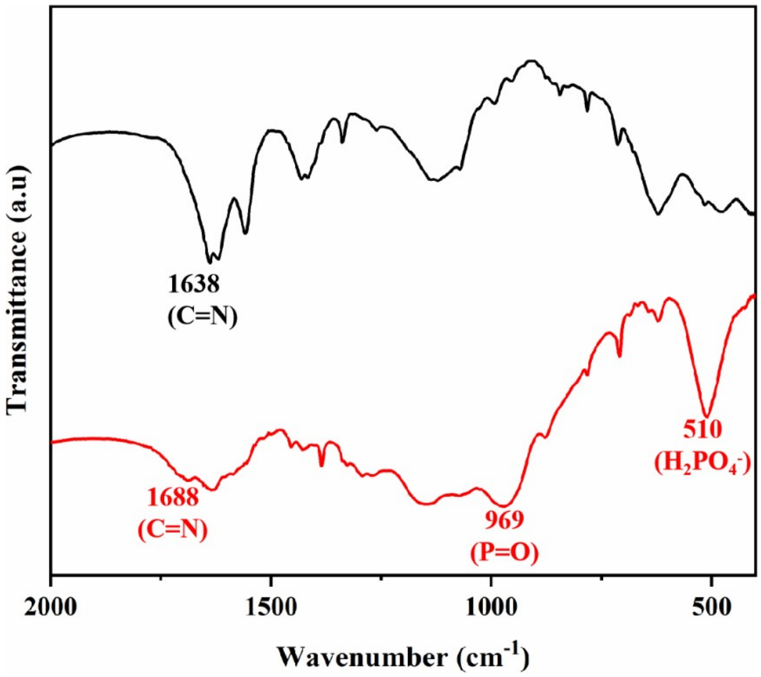

Figure S7: FT-IR spectra of **Tp-BI-COF** (black) and **H_3PO_4@Tp-BI-COF** (red).

Section S-5: Morphological study of Tp-BI-COF

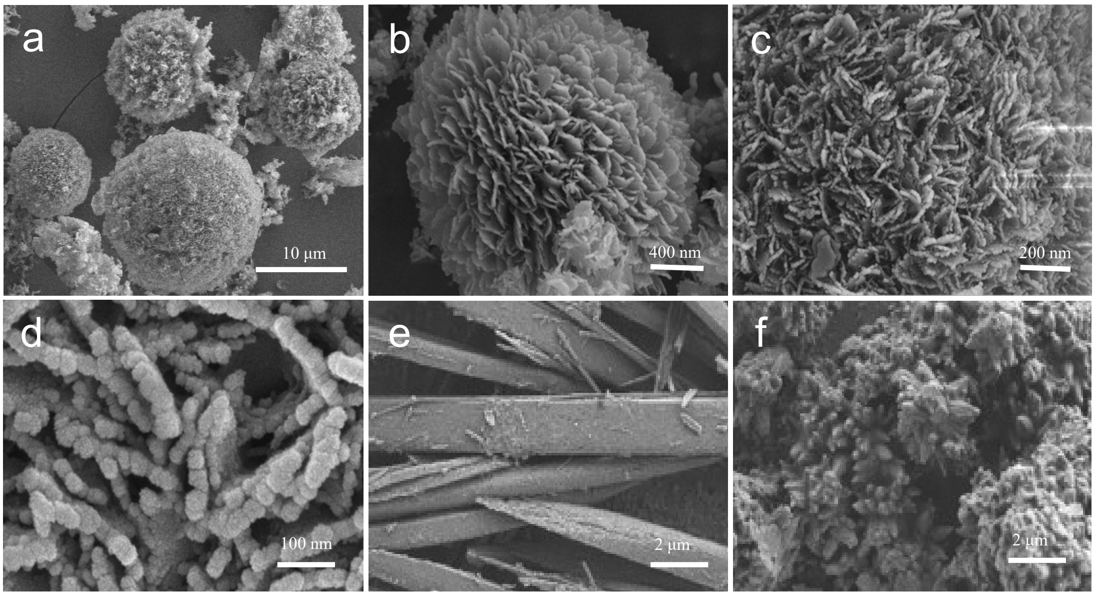

Figure S8: (a-d) SEM images of **Tp-BI-COF**, (e) SEM images of starting material **BI**, (f) SEM images of starting material **Tp**.

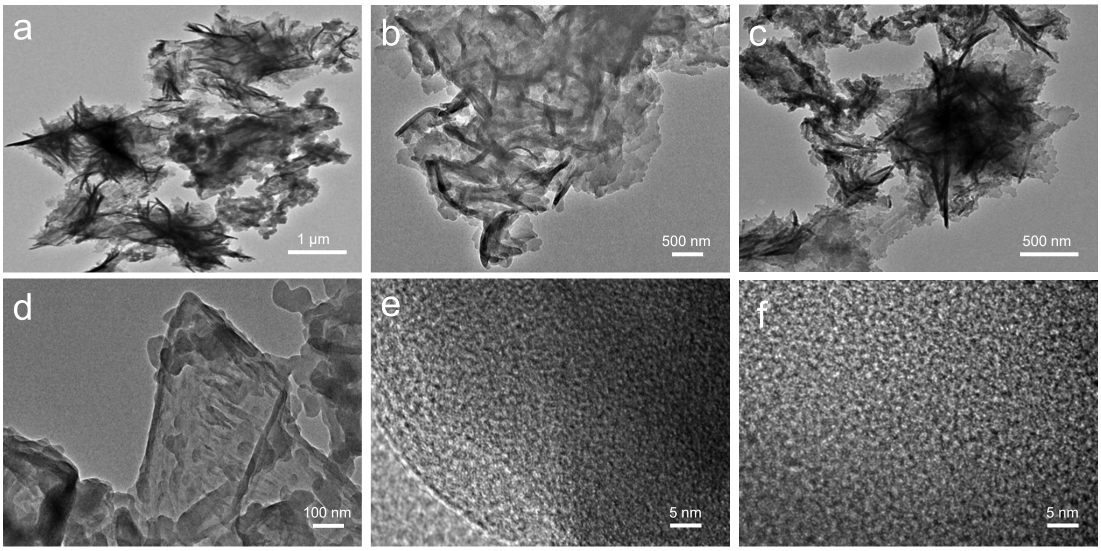

Figure S9: (a-f) HRTEM images of **Tp-BI-COF**.

Section S-6: Optimization of Tp-BI-COF Synthesis Scheme

Table S2. BET surface area of **Tp-BI-COF** synthesized by different experimental schemes.

serial number	solvent system (4 mL)	TEMP (°C)	HOAc (0.4 mL)	BET ($m^2\ g^{-1}$)
1	DMF	120	3 M	38.52
2	DMF: Mesitylene = 1: 1	120	3 M	53.43
3	DMF: 1,4-dioxane = 1: 1	120	3 M	34.12
4	1,4-dioxane: Mesitylene: 2,4,6-Trimethoxybenzaldehyde = 1: 1: 0.25	120	3 M	39.58
5	1,4-dioxane: Mesitylene = 5: 1	120	3 M	462.34
6	1,4-dioxane: Mesitylene = 3: 1	120	3 M	561.23
7	1,4-dioxane: Mesitylene = 1: 1	120	3 M	1018
8	1,4-dioxane: Mesitylene = 1: 3	120	3 M	752.86
9	1,4-dioxane: Mesitylene = 1: 5	120	3 M	698.12
10	1,4-dioxane: Mesitylene = 1: 7	120	3 M	489.67
11	1,4-dioxane: Mesitylene = 1: 9	120	3 M	539.83
12	1,4-dioxane: Mesitylene = 1: 1	120	1 M	110.57
13	1,4-dioxane: Mesitylene = 1: 1	120	2 M	980.71
14	1,4-dioxane: Mesitylene = 1: 1	120	5 M	982.48
15	1,4-dioxane: Mesitylene = 1: 1	120	6 M	982.67
16	1,4-dioxane: Mesitylene = 1: 1	90	3 M	576.32
17	1,4-dioxane: Mesitylene = 1: 1	100	3 M	612.94
18	1,4-dioxane: Mesitylene = 1: 1	110	3 M	783.68
19	1,4-dioxane: Mesitylene = 1: 1	130	3 M	893.15
20	1,4-dioxane: Mesitylene = 1: 1	140	3 M	912.58

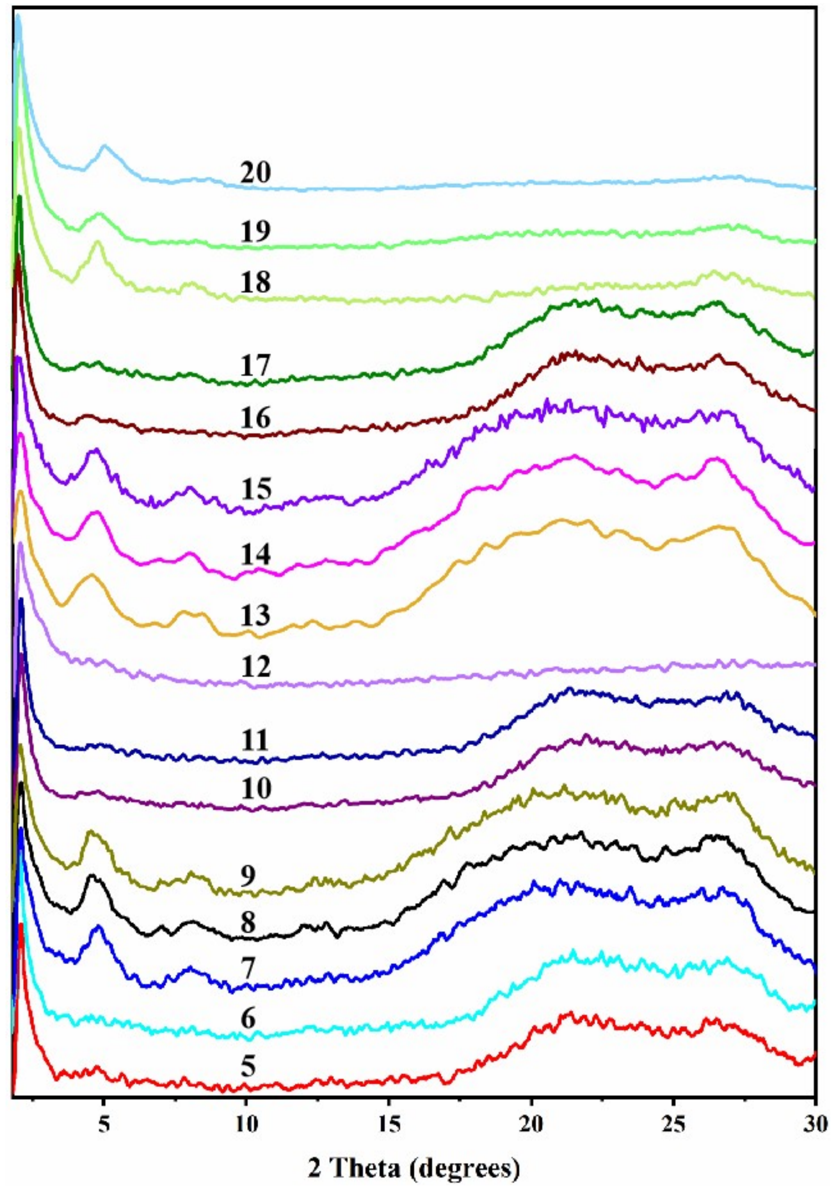

Figure S10: PXRD spectra of **Tp-BI-COF** synthesized with different conditions.

Section S-7: Gas Adsorption Studies

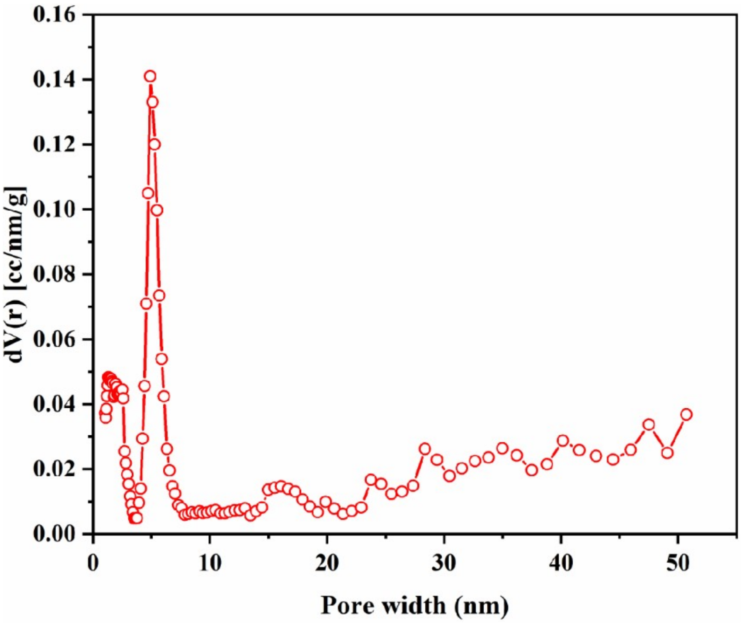

Figure S11: Pore size distribution of **Tp-BI-COF**, with a pore size of 4.9 nm.

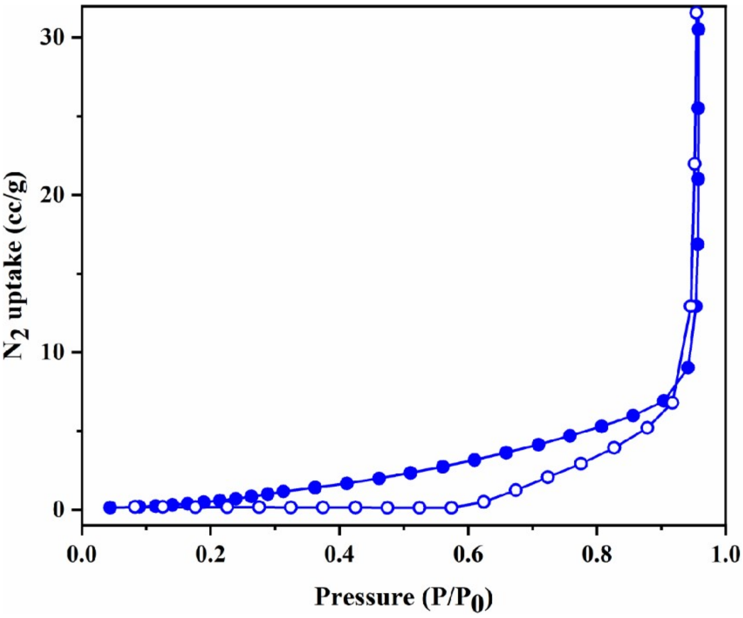

Figure S12: N_2 adsorption isotherm curves (77 K) of **H_3PO_4@Tp-BI-COF** (filled: adsorption, open: desorption).

Section S-8: Photochromic properties of Tp-BI-COF

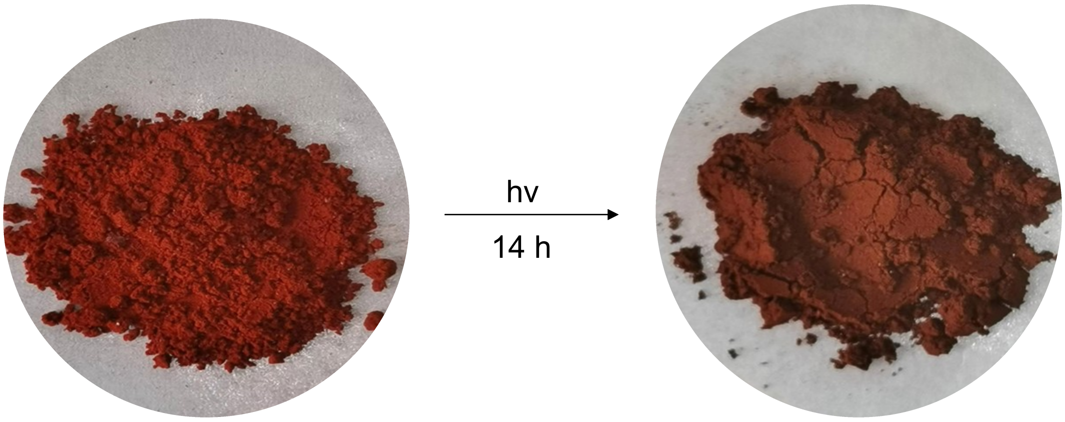

Figure S13: The color change photo of **Tp-BI-COF** before and after irradiation.

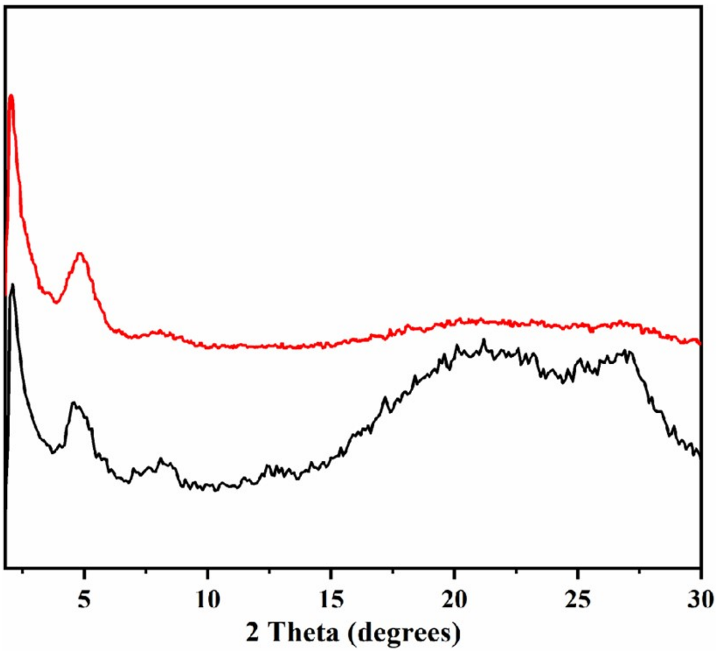

Figure S14: PXRD spectra of **Tp-BI-COF** before irradiation (black) and after irradiated for 14 h (red).

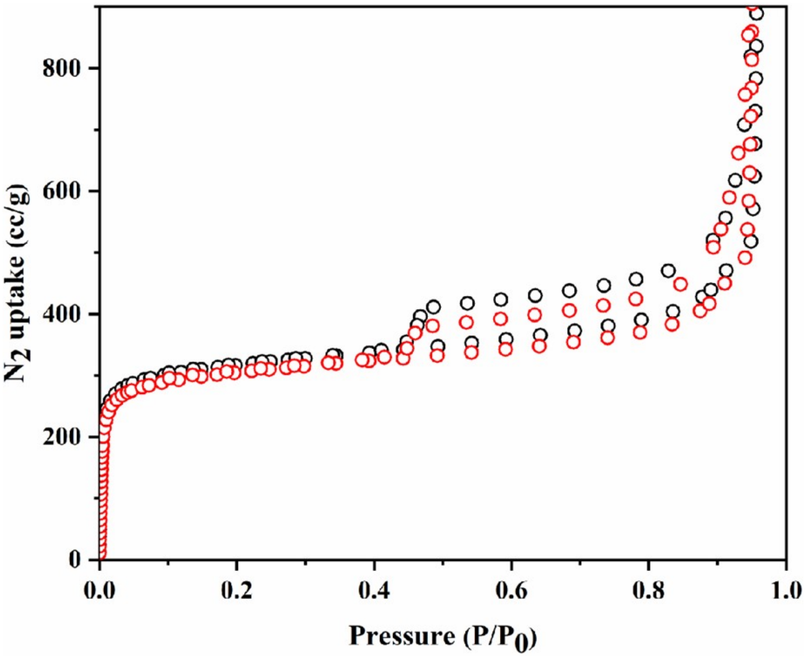

Figure S15: Nitrogen sorption isotherm profiles (77 K) of **Tp-BI-COF** before irradiation (black) and after irradiated for 14 h (red).

Section S-9: Preparation of H_3PO_4@Tp-BI-COF

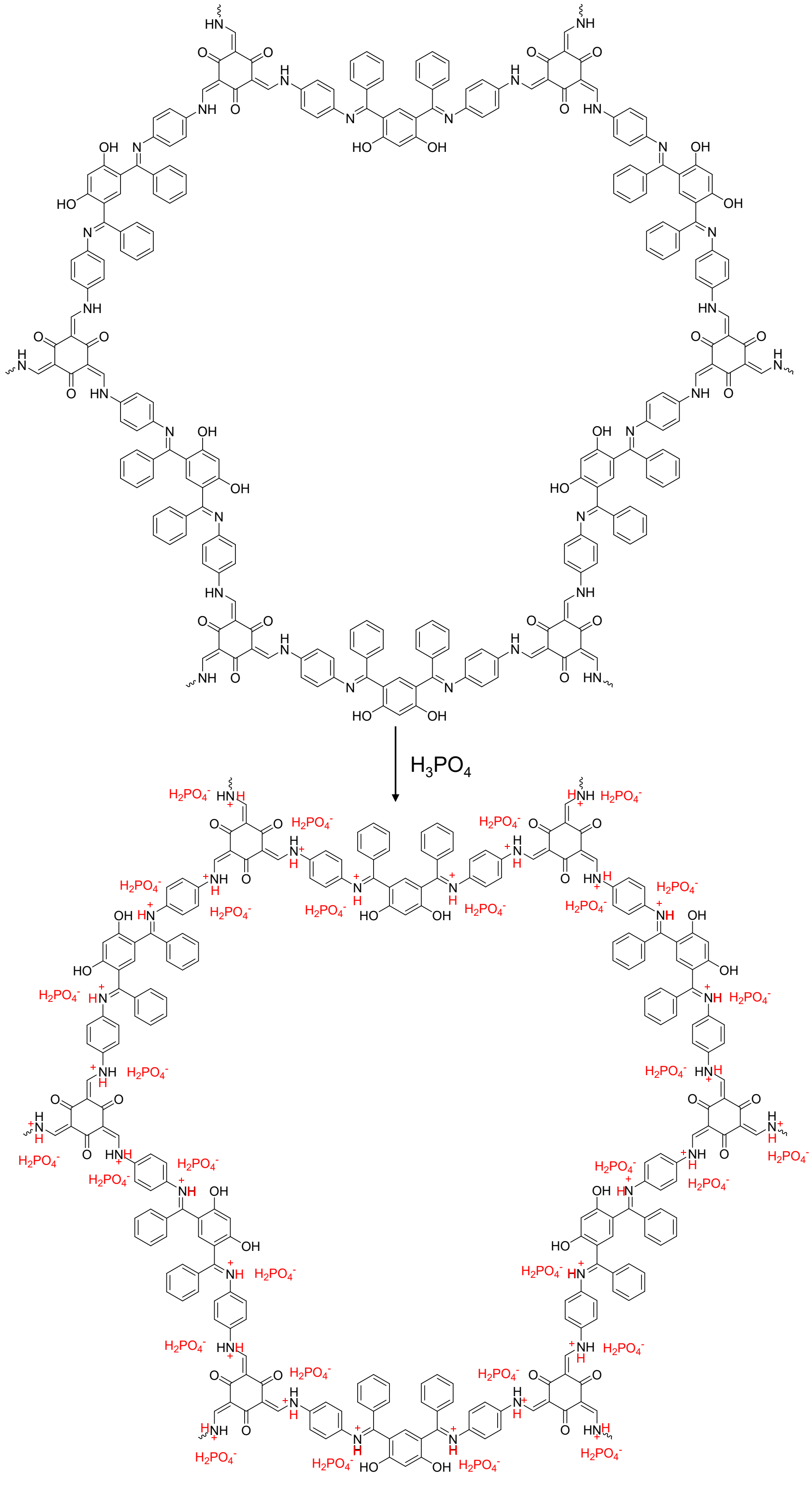

Figure S16: Schematic representation of protonation of **Tp-BI-COF**.

Section S-10: Stability study of Tp-BI-COF and H_3PO_4@Tp-BI-COF

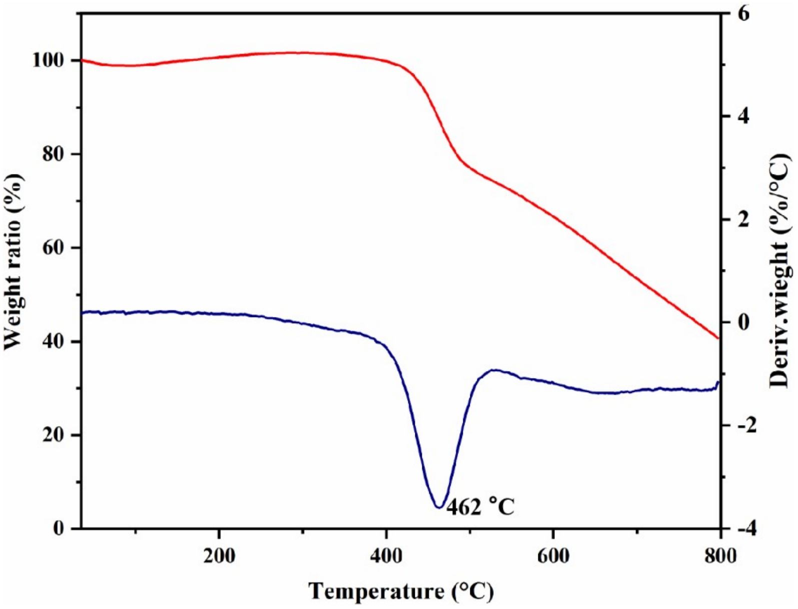

Figure S17: TGA data of **Tp-BI-COF** under N_2 atmosphere.

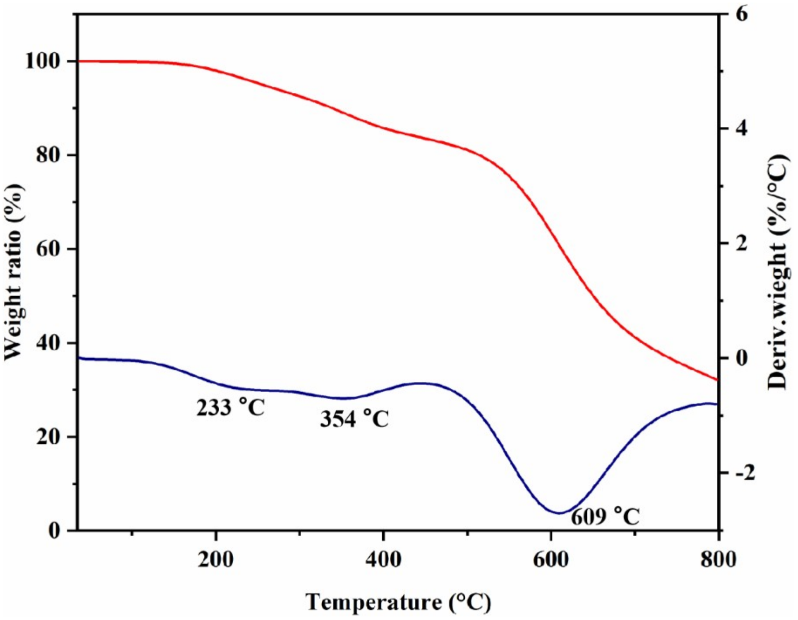

Figure S18: TGA data of **H_3PO_4@Tp-BI-COF** under N_2 atmosphere.

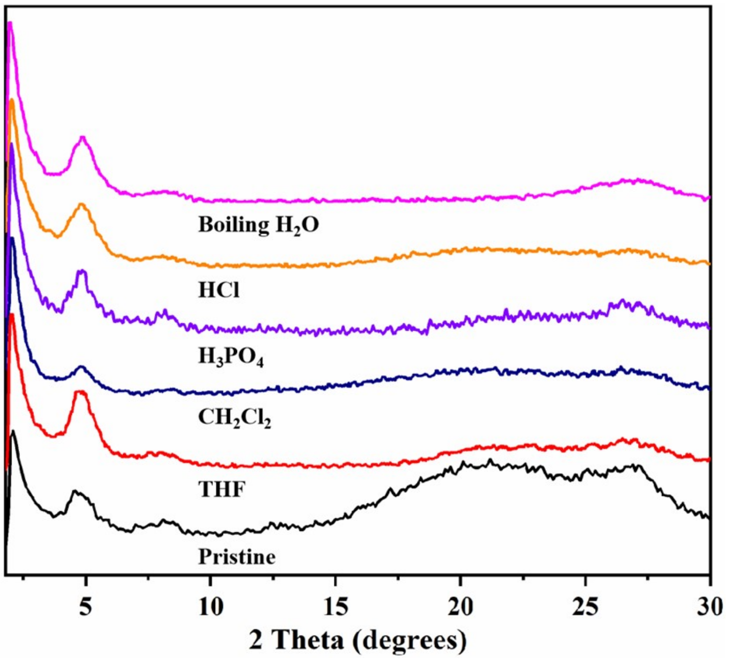

Figure S19: PXRD patterns of recovered **Tp-BI-COF** upon washing after immersed in different solvents for 7 days (black, pristine COF; red, THF; blue, CH_2Cl_2; violet, H_3PO_4; orange, HCl; pink, boiling water).

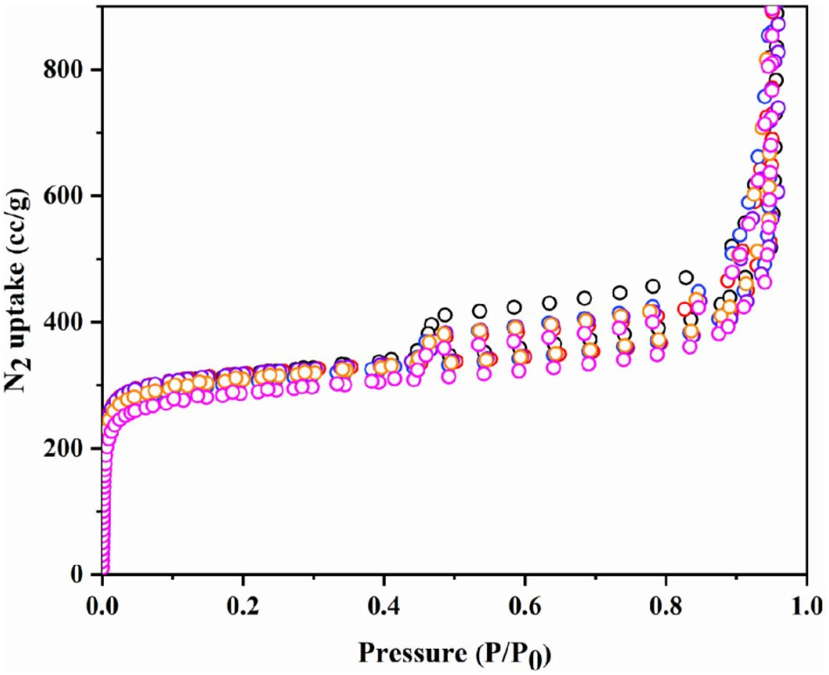

Figure S20: Nitrogen sorption isotherm profiles of recovered **Tp-BI-COF** measured at 77 K after treatment in different solvents for 7 days (black, pristine COF; red, THF; blue, CH_2Cl_2; violet, H_3PO_4; orange, HCl; pink, boiling water).

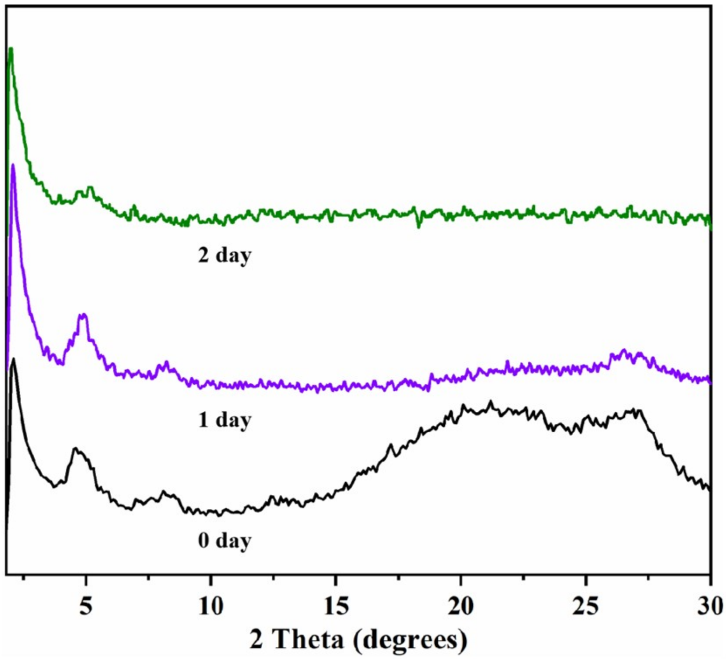

Figure S21: PXRD patterns of recovered **Tp-BI-COF** upon washing with H_2O after immersing in aqueous NaOH (9 M) for different time (black, 0 day; violet, 1 day; green, 2 day).

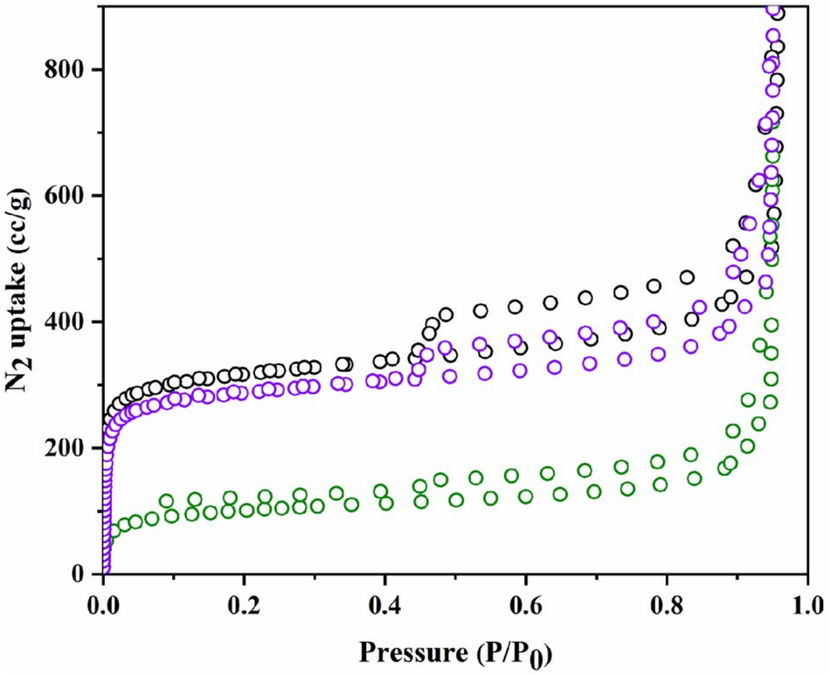

Figure S22: Nitrogen sorption isotherm profiles of recovered **Tp-BI-COF** measured at 77 K after treatment in aqueous NaOH (9 M) for different time (black, 0 day; violet, 1 day; green, 2 day).

Section S-11: PXRD study of Tp-BI-COF and H_3PO_4@Tp-BI-COF

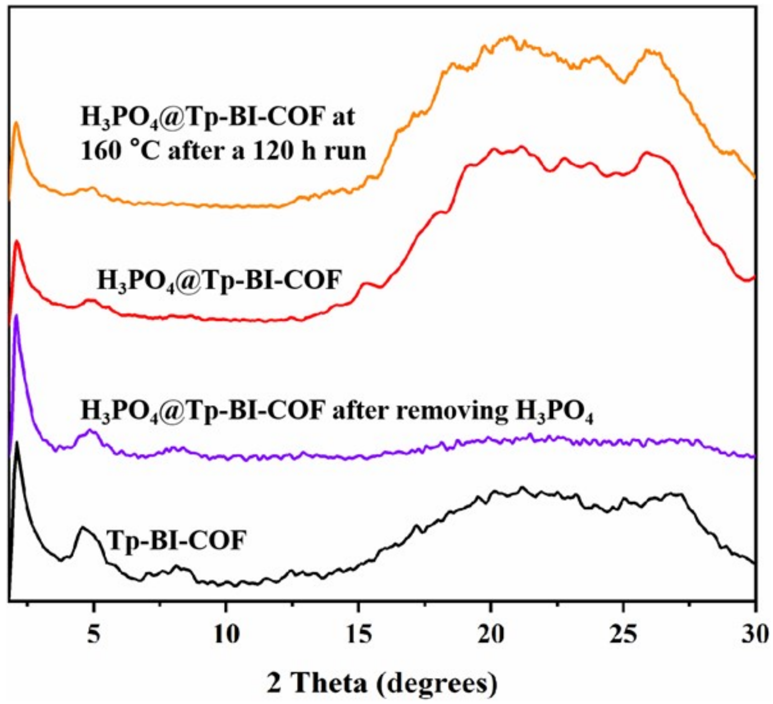

Figure S23: PXRD spectra of **Tp-BI-COF** (black), **H_3PO_4@Tp-BI-COF** after removing H_3PO_4 (purple), **H_3PO_4@Tp-BI-COF** (red) and **H_3PO_4@Tp-BI-COF** after running at 160 ℃ for 120 h (orange).

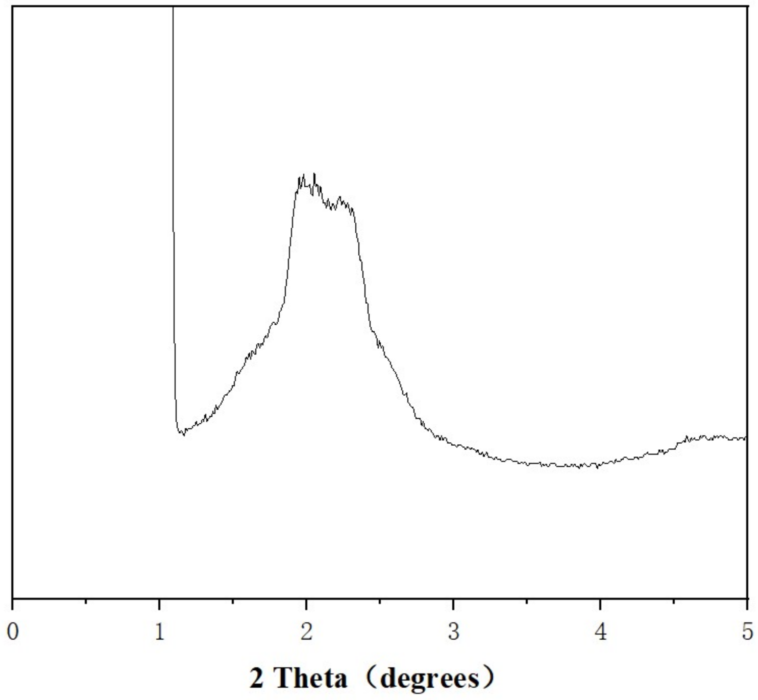

Figure S24: SAXS of **Tp-BI-COF**.

Section S-12: Energy dispersive X-ray spectroscopy

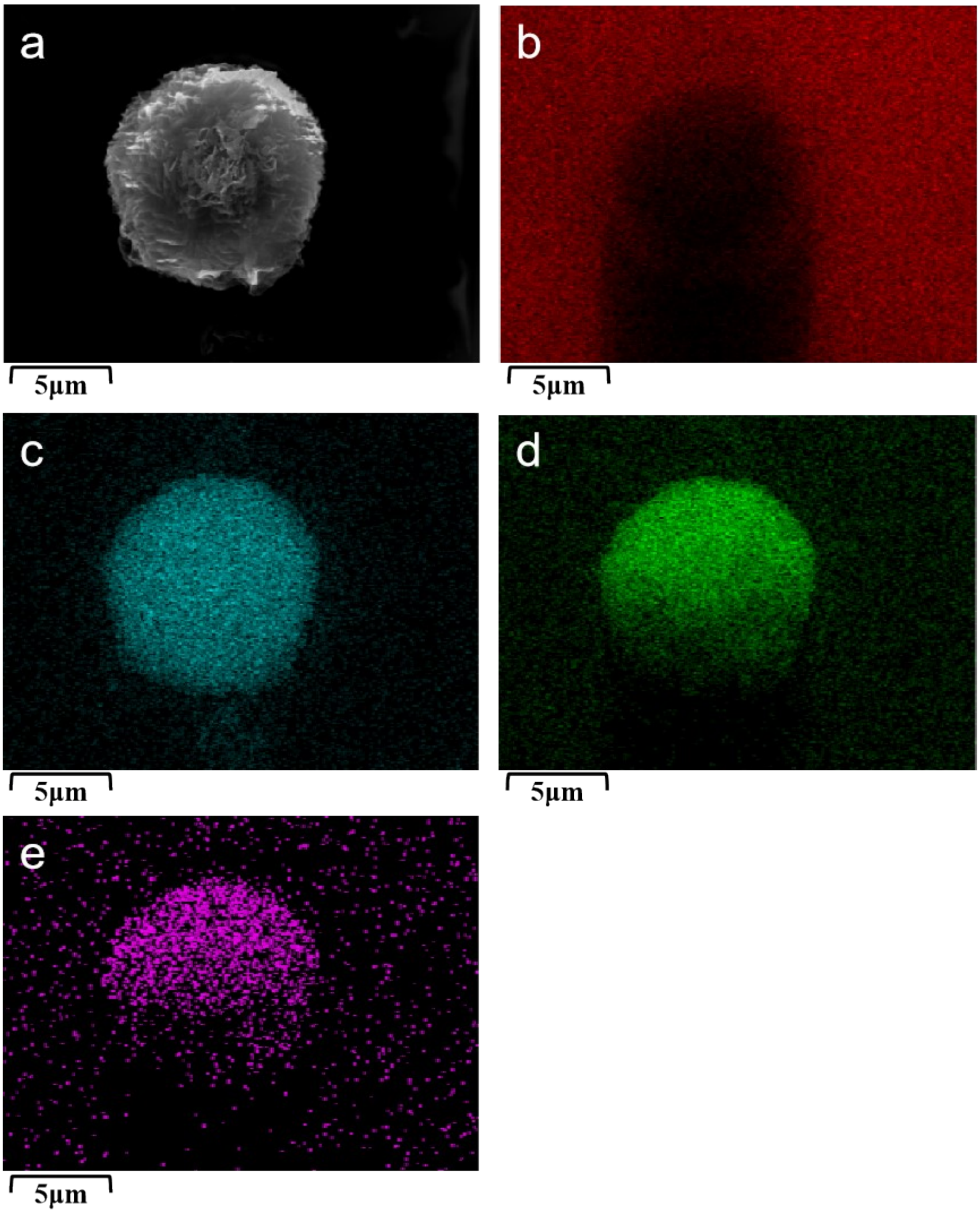

Figure S25: Energy dispersive X-ray spectroscopy. (a) SEM image of **H_3PO_4@Tp-BI-COF**. (b-e) Elemental distributions of (b) carbon, (c) phosphorus, (d) oxygen, and (e) nitrogen.

Section S-13: High-resolution XPS spectra

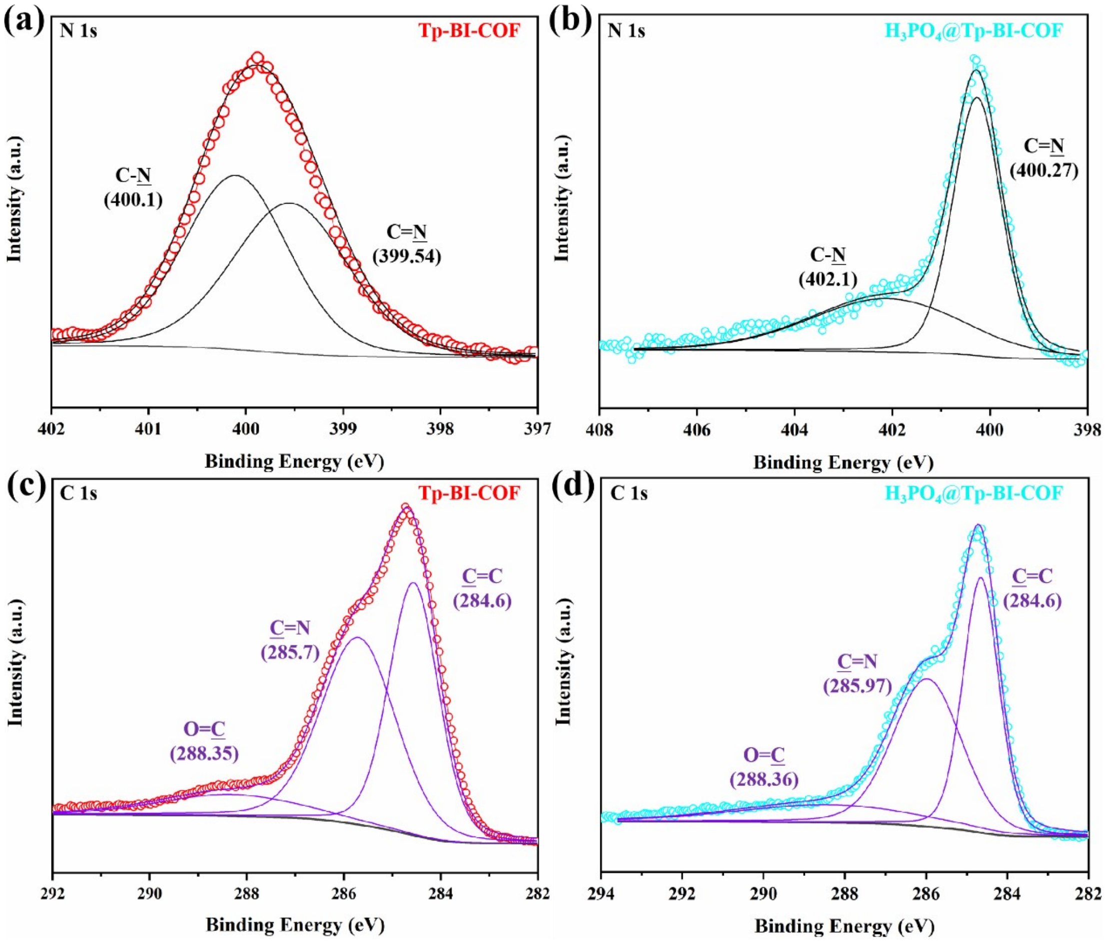

Figure S26: High-resolution XPS spectra of the (a) N 1s band of **Tp-BI-COF**, (b) N 1s band of **H_3PO_4@Tp-BI-COF**, (c) C 1s band of **Tp-BI-COF**, and (d) C 1s band of **H_3PO_4@Tp-BI-COF**.

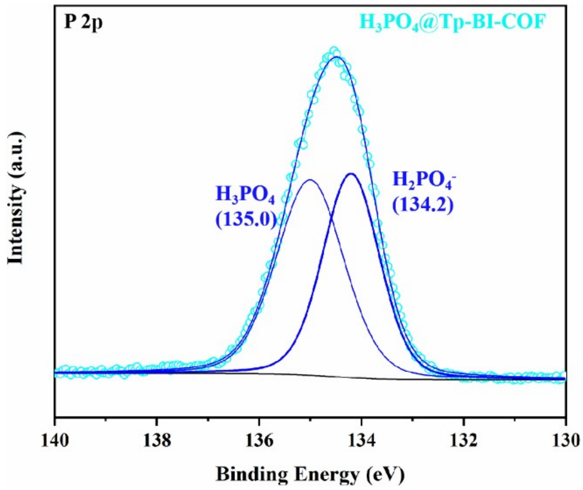

Figure S27: High-resolution P 2p XPS spectra of **H_3PO_4@Tp-BI-COF**.

Section S-14: Nyquist plots of H_3PO_4@Tp-BI-COF

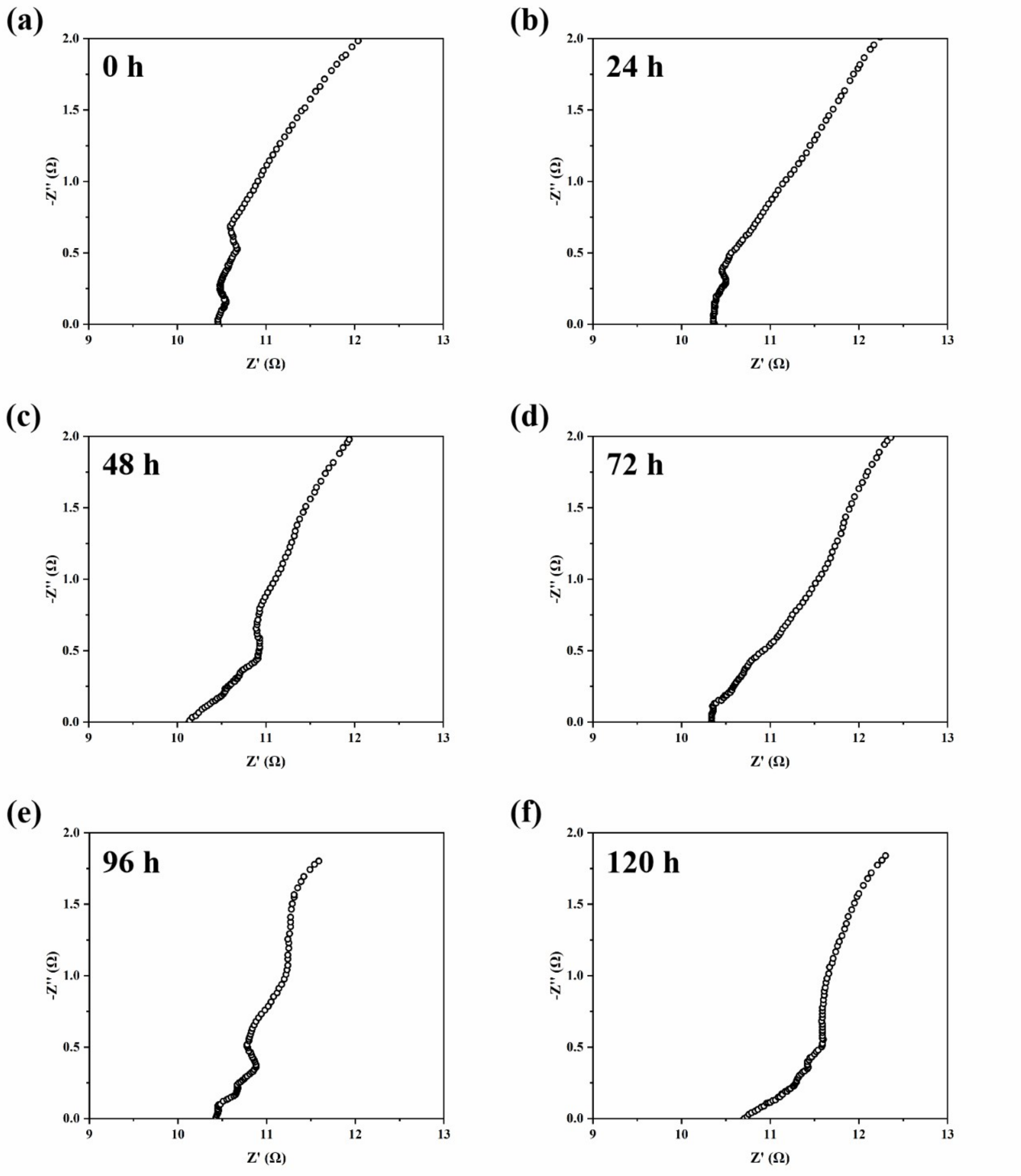

Figure S28: Nyquist plots of **H_3PO_4@Tp-BI-COF** at 160 °C: (a) 0 hour, (b) 24 hours, (c) 48 hours, (d) 72 hours, (e) 96 hours, and (f) 120 hours. (sample weight: 148 mg; pellet radius: 6.5 mm; pellet thickness: 0.8 mm).

Section S-15: Current status of anhydrous proton conductors

Table S3. Summary of the state-of art anhydrous proton conductors based on COFs

Materials	σ (S cm^{-1})	T (K)	Ref.
H_3PO_4@Tp-BI-COF	5.95×10^{-3}	433	**This work**
H_3PO_4@Tp-Azo-COF	6.70×10^{-5}	340	Ref.[5]
H_3PO_4@TpBpy-MC	2.50×10^{-3}	393	Ref.[6]
H_3PO_4@TpBpy-ST	1.98×10^{-3}	393	Ref.[6]
TpPa-SO_3H	1.70×10^{-5}	393	Ref.[7]
Phytic acid@TpPa-(SO_3H-Py)	5.00×10^{-4}	393	Ref.[7]
aza-COF-2_H	4.80×10^{-3}	323 (97% RH)	Ref.[8]
LiCl@RT-COF-1	6.45×10^{-3}	313 (100% RH)	Ref.[9]
Im@TPB-DMTP-COF	4.37×10^{-3}	403	Ref.[10]
Tri@TPB-DMTP-COF	1.10×10^{-3}	403	Ref.[10]
Im@Td-PPI	3.49×10^{-4}	363	Ref.[11]
Im@Td-PNDI	9.04×10^{-5}	363	Ref.[11]

Section S-16: References

1. J. H. Chong, M. Sauer, B. O. Patrick and M. J. MacLachlan, *Org. Lett.*, 2003, **5**, 3823.
2. M. Abdul-Aziz, J. V. Auping and M. A. Meador, *J. Org. Chem.*, 1995, **60**, 1303.
3. (a) Y. Zhu, H. Yang, Y. Jin and W. Zhang, *Chem. Mater.,* 2013, **25**, 3718; (b) W. Lu, D.Yuan, D. Zhao, C.I. Schilling, O. Plietzsch, T. Muller, S. Bras̆e, J. Guenther, J. Blümel, R. Krishna, Z. Li and H.C. Zhou, *Chem. Mater*., 2010, **22**, 5964; (c) J.X. Jiang, F. Su, A. Trewin, C. D. Wood, N. L. Campbell, H. Niu, C. Dickinson, A. Y. Ganin, M. J. Rosseinsky, Y. Z. Khimyak and A. I. Cooper, *Angew. Chem. Int. Ed.,* 2007, **46**,8574.
4. S. Tao, L. Zhai, A. D. D. Wonanke, M. A. Addicoat, Q. Jiang and D. Jiang, *Nat. Commun.*, 2020, **11**, 1981.
5. S. Chandra, T. Kundu, S. Kandambeth, R. Babarao, Y. Marathe, S. M. Kunjir and R. Banerjee, *J Am Chem Soc*, 2014, **136**, 6570.
6. D. B. Shinde, H. B. Aiyappa, M. Bhadra, B. P. Biswal, P. Wadge, S. Kandambeth, B. Garai, T. Kundu, S. Kurungot and R. Banerjee, *Journal of Materials Chemistry A*, 2016, **4**, 2682.
7. S. Chandra, T. Kundu, K. Dey, M. Addicoat, T. Heine and R. Banerjee, *Chemistry of Materials*, 2016, **28**, 1489.
8. Z. Meng, A. Aykanat and K. A. Mirica, *Chemistry of Materials*, 2018, **31**, 819.
9. C. Montoro, D. Rodriguez-San-Miguel, E. Polo, R. Escudero-Cid, M. L. Ruiz-Gonzalez, J. A. R. Navarro, P. Ocon and F. Zamora, *J Am Chem Soc*, 2017, **139**, 10079.
10. H. Xu, S. Tao and D. Jiang, *Nat Mater*, 2016, **15**, 722.
11. Y. Ye, L. Zhang, Q. Peng, G. E. Wang, Y. Shen, Z. Li, L. Wang, X. Ma, Q. H. Chen, Z. Zhang and S. Xiang, *J Am Chem Soc*, 2015, **137**, 913.
